# Capsaicin for cardiometabolic syndrome: multitarget mechanisms and therapeutic potential

**DOI:** 10.3389/fnut.2026.1771003

**Published:** 2026-01-30

**Authors:** Jinyuan Lin, Honglei Shen, Huajin Ou, Huilin Luo, Dongqin Huang, Liu Ye

**Affiliations:** 1 Department of Anesthesiology, Guangxi Medical University Cancer Hospital, Nanning, China; 2 Guangxi Engineering Research Center for Tissue & Organ Injury and Repair Medicine, Nanning, China; 3 Guangxi Health Commission Key Laboratory of Basic Science and Prevention of Perioperative Organ Disfunction, Nanning, China; 4 Guangxi Clinical Research Center for Anesthesiology, Nanning, China

**Keywords:** capsaicin, cardiometabolic syndrome, energy metabolism, gut microbiota, inflammation, insulin resistance, lipid metabolism, TRPV1

## Abstract

Cardiometabolic syndrome (CMS) is a multifactorial disorder characterized by the clustering of central obesity, insulin resistance, atherogenic dyslipidemia, hypertension, and chronic low-grade inflammation, collectively predisposing individuals to type 2 diabetes and increased cardiovascular morbidity and mortality. Capsaicin, the principal bioactive compound derived from chili peppers, has attracted growing interest as a multitarget modulator of the complex pathophysiology underlying CMS. Accumulating evidence indicates that capsaicin confers cardiometabolic protection predominantly through transient receptor potential vanilloid 1 (TRPV1)-mediated signaling, while additional TRPV1-independent mechanisms may also contribute. These actions include enhancement of energy metabolism, improvement of insulin sensitivity, suppression of inflammatory and oxidative pathways, regulation of lipid homeostasis, and preservation of vascular function. Recent studies highlight the importance of a capsaicin–gut microbiota axis, whereby capsaicin reshapes microbial composition, modulates bile acid and short-chain fatty acid signaling, and reinforces intestinal barrier integrity, thereby exerting systemic metabolic and cardiovascular benefits. Despite compelling mechanistic and preclinical evidence, translation to clinical application remains limited by variability in effective dosing, bioavailability, and interindividual differences in gut microbiota composition. This review synthesizes current advances in the molecular and physiological actions of capsaicin and discusses future perspectives for its clinical development as an adjunctive strategy for CMS management.

## 1 Introduction

Cardiometabolic syndrome (CMS) is defined by the clustering of central obesity, insulin resistance, atherogenic dyslipidemia, hypertension, and chronic low-grade inflammation, a constellation that markedly increases the risk of type 2 diabetes, cardiovascular disease, and premature mortality (1). With the continuing global rise in obesity and metabolic disorders, CMS has emerged as a major public health challenge, placing a substantial burden on healthcare systems worldwide (2). Although lifestyle modification and pharmacologic control of risk components such as hyperglycemia, dyslipidemia, and hypertension have improved outcomes, these strategies remain largely single-targeted (3). These single-targeted approaches are insufficient to address the complex and tightly integrated pathophysiology of CMS. This gap highlights the urgent need for multi-target strategies capable of simultaneously modulating metabolic, inflammatory, and vascular dysfunction (1, 4).

**Figure 1:**
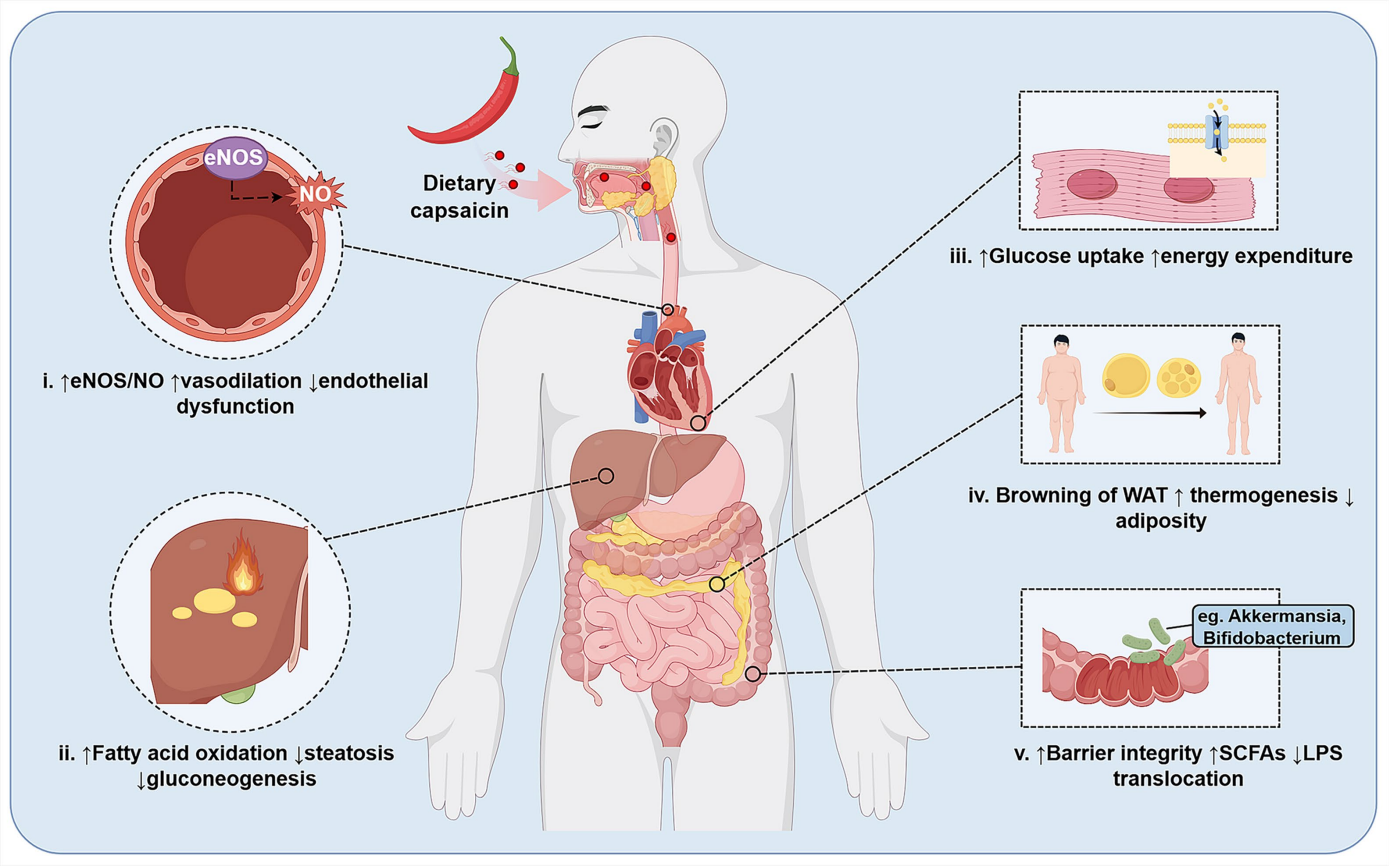
Organ-level actions of dietary capsaicin relevant to metabolic and cardiovascular regulation. Following ingestion, dietary capsaicin acts on multiple organs to improve cardiometabolic homeostasis: (i) vasculature: capsaicin increases endothelial eNOS activity and NO production, leading to enhanced vasodilation and attenuation of endothelial dysfunction; (ii) liver: capsaicin promotes fatty acid oxidation and reduces steatosis and gluconeogenesis, thereby improving hepatic metabolic status; (iii) skeletal muscle: capsaicin enhances insulin-stimulated glucose uptake and contributes to increased whole-body energy expenditure; (iv) adipose tissue: capsaicin drives browning of white adipose tissue, augments thermogenesis, and reduces adiposity; (v) gut: capsaicin strengthens epithelial barrier integrity, increases short-chain fatty acids (SCFAs), lowers LPS translocation, and selectively enriches beneficial taxa such as *Akkermansia* and *Bifidobacterium*. Together, these coordinated organ-specific effects support improved vascular function, energy balance, and systemic inflammatory and metabolic profiles.

Capsaicin, a pungent vanilloid compound derived from chili peppers, has gained increasing attention as a bioactive molecule with broad cardiometabolic relevance. It exerts its biological effects through both transient receptor potential vanilloid 1 (TRPV1) channel-dependent and -independent activation. Beyond its well established sensory and analgesic properties, capsaicin enhances sympathetic activity and stimulates thermogenesis in brown and beige adipose tissue as well as skeletal muscle via TRPV1 activation-induced Ca^2+^ dependent signaling cascades, leading to increased whole-body energy expenditure, reduced adiposity, and improved lipid profiles (5–7). This effect ameliorates metabolic risk markers in individuals with obesity or metabolic syndrome, highlighting its therapeutic relevance to human cardiometabolic health (8, 9). At the intracellular level, capsaicin engages key metabolic regulators, including 5′-AMP-activated protein kinase (AMPK) and peroxisome proliferator-activated receptor-γ coactivator-1α (PGC-1α), which coordinate glucose uptake and oxidation, fatty acid β-oxidation, suppression of *de novo* lipogenesis, and mitochondrial biogenesis in liver, muscle, and adipose tissue (10–12). In parallel, capsaicin modulates insulin signaling and suppresses inflammatory pathways involving nuclear factor kappa B (NF-κB) and the NLRP3 inflammasome, which in turn regulate adipokine secretion and adipose tissue browning, improve endothelial and cardiac function, and influence gut microbiota–derived metabolites and bile acid signaling pathways (4, 13–15). Collectively, these beneficial actions converge on multiple pathogenic nodes of CMS (16–18). (Figure 1).

Despite these advances, an integrated framework explaining how capsaicin orchestrates its multitarget actions across metabolic and cardiovascular systems in CMS remains incomplete. This review therefore aims to systematically synthesize and critically evaluate current evidence regarding the cardiometabolic effects of capsaicin and TRPV1 signaling. We summarize how capsaicin regulates energy metabolism through TRPV1 activation, AMPK signaling, and mitochondrial biogenesis and optimization. We further examine its roles in alleviating insulin resistance, suppressing inflammatory and oxidative pathways, reshaping lipid metabolism and cardiovascular risk, and modulating the emerging capsaicin–gut microbiota axis. By integrating mechanistic, preclinical, and clinical evidence, this review seeks to position capsaicin as a plausible natural multitarget therapeutic candidate and to provide a conceptual framework for future interventions targeting the complex pathophysiology of CMS.

This narrative review is based on a focused survey of the literature, prioritizing high-quality experimental and clinical studies relevant to CMS. Efforts were made to ensure balanced coverage of mechanistic, preclinical, and clinical evidence.

### 1.1 Mechanisms of capsaicin regulating energy metabolism

#### 1.1.1 TRPV1-dependent and -independent energy expenditure

Capsaicin-induced thermogenesis in adipose tissue is initiated by activation of TRPV1, a Ca^2+^ permeable cation channel whose opening elevates intracellular Ca^2+^ and triggers downstream kinase signaling (5–7). In brown and beige adipocytes, TRPV1 activation induces a thermogenic transcriptional program. This program involves sirtuin 1 (SIRT1) dependent deacetylation of PR domain containing 16 (PRDM16), which enhances its coactivator activity and induces uncoupling protein 1 (UCP1) expression. Increased UCP1 uncouples oxidative phosphorylation and dissipates the proton gradient as heat (5, 6). In mice with diet induced obesity, dietary capsaicin supplementation or genetic activation of TRPV1 increases the number of UCP1 positive beige adipocytes in white adipose tissue and improves resistance to obesity. In contrast, deletion of TRPV1, UCP1, or PRDM16 in adipose tissue abolishes these effects, linking this pathway to whole body energy expenditure and adiposity control (5, 7). Consistent with these findings, capsaicin increases UCP1 expression, mitochondrial biogenesis, and oxygen consumption in human beige adipocytes. These results indicate that the TRPV1 dependent thermogenic program is conserved in human adipose tissue (19). Collectively, these findings establish a TRPV1-dependent thermogenic program in adipose tissue, providing a mechanistic framework for interpreting human studies in which dietary capsaicin or capsinoids modestly increase energy expenditure, particularly in individuals with metabolically active brown adipose tissue (20–23).

In contrast to adipose tissue, capsaicin engages a complementary thermogenic mechanism in skeletal muscle that is largely independent of both UCP1 and direct TRPV1 activation. In C2C12 myotubes and skeletal muscle from obese mice, capsaicin upregulates sarco/endoplasmic reticulum Ca^2+^-ATPases (SERCA1/2) and ryanodine receptors (RYR1/2), thereby activating ATP-consuming Ca^2+^ futile cycles that recycle Ca^2+^ across the sarcoplasmic reticulum and dissipate energy as heat (13). Capsaicin also increases the expression of creatine kinase B and mitochondrial creatine kinase 2, supporting a creatine-driven substrate cycle that accelerates mitochondrial ATP turnover and thermogenesis (13). Mechanistic analyses indicate that these Ca^2+^ and creatine futile cycles are recruited in obese mice primarily through coordinated activation of α₁, β₂ and β₃ adrenergic receptors. TRPV1 acts as a synergistic, but not essential, contributor to this process. This pathway provides a muscle-based thermogenic route that operates largely independently of UCP1 (13). These observations align with broader evidence identifying creatine dependent substrate cycling and SERCA mediated Ca^2+^ cycling as major thermogenic mechanisms in beige and brown adipocytes. Loss of either pathway markedly reduces thermogenic capacity (24–27).

Collectively, these cellular and molecular mechanisms underpin clinical studies of non-pungent capsaicin analogs (capsinoids). Acute capsinoid ingestion selectively increases whole body energy expenditure in individuals with metabolically active brown adipose tissue, as assessed by ^18^F-FDG PET/CT, indicating that brown adipose tissue (BAT) mediates the thermogenic response to capsinoids (20, 21). Longer term capsinoid supplementation increases brown adipose tissue vascular density and resting energy expenditure in healthy middle-aged adults. Meta analyses of clinical trials also show modest but significant increases in resting metabolic rate and fat oxidation, particularly in individuals with overweight or obesity (22, 23). These findings support the concept that capsaicin and related compounds can enhance human energy expenditure, primarily through activation of brown and beige adipose thermogenesis, with TRPV1 serving as a key but context-dependent mediator.

#### 1.1.2 Regulation of AMPK signaling pathway to promote metabolic homeostasis

Beyond its direct thermogenic actions, capsaicin promotes metabolic homeostasis by engaging the AMPK signaling axis downstream of TRPV1/Ca^2+^. AMPK functions as a central energy sensor that restores metabolic balance by coordinating glucose and lipid fluxes. Mechanistic studies indicate that capsaicin activates AMPK through cell specific upstream mechanisms. These include calcium/calmodulin-dependent protein kinase kinase 2 (CaMKK2, also known as CaMKKβ) (28), a TRPV1/Ca^2+^/CaMKK2 axis in skeletal muscle cells (29), and a TRPV1/liver kinase B1 (LKB1) pathway in prostate cancer cells (30). Together, these pathways define a context dependent AMP activated protein kinase activation network linked to TRPV1.

In hepatic lipid homeostasis, capsaicin and capsaicinoid derivatives shift metabolism toward oxidation and clearance. In LKB1-deficient HepG2 hepatocytes challenged with oleic acid, capsaicin or capsaicinoid glucoside increases AMPK phosphorylation via a TRPV1/Ca^2+^/CaMKK2 dependent mechanism, leading to repression of the lipogenic transcription factor sterol regulatory element binding protein 1c (SREBP-1c) and its downstream targets, including fatty acid synthase (FASN) and acetyl-CoA carboxylase (ACC) (10–12). In parallel, AMPK mediated inhibition of ACC reduces malonyl CoA levels and relieves inhibition of carnitine palmitoyl transferase 1 (CPT1), facilitating mitochondrial β-oxidation and fatty-acid catabolism (12). These coordinated actions suppress lipogenesis and promote lipid oxidation, accounting for reduced triglyceride and total cholesterol accumulation in oleic acid treated LKB1-deficient HepG2 cells exposed to capsaicin or capsaicinoid glucoside (10–12). Consistent with cellular findings, chronic administration of Miao sour soup, a fermented food containing capsaicin, lycopene, and organic acids, reduces weight gain and adiposity in obese rats fed a high fat diet. It improves plasma lipid profiles, increases hepatic AMPK alpha expression, and reduces SREBP 1c, ACC alpha, and FASN levels (11).

AMPK activation by capsaicin also favorably remodels glucose utilization. In differentiated C2C12 skeletal muscle cells, TRPV1 mediated Ca^2+^ entry activates CaMKK2 and AMPK, enhancing glucose oxidation and ATP production through insulin independent mechanisms. Pharmacological inhibition of TRPV1 or CaMKK2 reduces AMPK phosphorylation, glucose oxidation and ATP generation. This mechanism explains how capsaicin bypass impaired insulin receptor substrate-1 (IRS-1)/phosphatidylinositol 3-kinase (PI3K)/protein kinase B (Akt) signaling to maintain cellular energy supply in skeletal muscle (29). In primary hepatocytes, capsaicin accelerates glucose uptake and utilization while increasing ATP production. These effects are associated with increased intracellular Ca^2+^ and transcriptional regulation of genes involved in glucose and amino acid metabolism (31).

Beyond classical metabolic tissues, AMPK signaling activated by capsaicin, contributes to broader cardiometabolic protection. In endothelial cells exposed to intermittent hyperglycemia, capsaicin activates a TRPV1/[Ca^2+^]i/CaMK2/AMPK pathway that upregulates SIRT1 and suppresses oxidative stress induced senescence, thereby preserving endothelial function (32). In the hypothalamic paraventricular nucleus of salt sensitive hypertensive rats, capsaicin pretreatment attenuates blood pressure through AMPK/Akt/nuclear factor erythroid 2 related factor 2 (Nrf2) signaling, accompanied by reduced neuroinflammation and autonomic dysregulation (33). In cardiomyocytes, TRPV1 activation mitigates hypoxic injury by improving autophagic flux via AMPK signaling, thereby supporting mitochondrial integrity and cell survival (34). In cancer models, capsaicin engages AMPK/AKt/mTOR or AMPK/mTOR pathways to suppress the Warburg effect, inhibit lipogenesis, promote mitochondrial dependent apoptosis, and induce autophagy, collectively restraining malignant phenotypes such as proliferation, migration, invasion, and epithelial mesenchymal transition (30, 35, 36).

These findings identify AMPK as a central downstream effector of TRPV1/Ca^2+^ signaling across multiple tissues. By inhibiting lipogenesis, enhancing fatty acid oxidation, improving glucose utilization, and supporting vascular, neural, cardiac, and cellular metabolic stability, capsaicin-activated AMPK represents a key mechanism by which capsaicin promotes metabolic homeostasis under conditions of nutrient excess and cardiometabolic stress.

#### 1.1.3 Promoting mitochondrial biogenesis and functional optimization

Building on TRPV1/AMPK/SIRT1 signaling, capsaicin further enhances metabolic resilience by stimulating mitochondrial biogenesis and optimizing mitochondrial function across multiple organs. A well characterized example is observed in a high fat diet model of obesity induced metabolic dysfunction-associated steatotic liver disease (MASLD), where dietary capsaicin activates hepatic TRPV1 and increases intracellular Ca^2+^ and AMPK phosphorylation and drives SIRT1-dependent upregulation of PPARα and the mitochondrial biogenesis coactivator PGC-1α. This transcriptional program increases hepatic mitochondrial biogenesis and fatty acid oxidation, thereby strengthening mitochondrial oxidative capacity under chronic metabolic overload (37). Increased expression of mitochondrial structural and respiratory proteins, including cytochrome c oxidase subunit IV (COX IV) and voltage-dependent anion channel 1 (VDAC1), further supports expansion of the oxidative machinery (38). Consistently, in HepG2 hepatocytes, capsaicin activates AMPK, increases PGC-1α abundance, improves mitochondrial function, and suppresses lipogenesis, linking mitochondrial biogenesis to coordinated control of lipid metabolism (10).

Capsaicin also preserves mitochondrial function under conditions of severe cellular stress. In septic acute liver injury, capsaicin attenuates mitochondrial dysfunction by limiting reactive oxygen species (ROS) generation, stabilizing mitochondrial membrane potential, preserving electron transport chain activity, and reducing cytochrome c release and apoptosis, thereby sustaining oxidative phosphorylation and ATP production (39). Similar protective effects are observed in cardiomyocytes exposed to anoxia or anoxia/reoxygenation, where capsaicin upregulates 14-3-3η, maintains mitochondrial membrane potential, improves respiratory complex function, and suppresses apoptotic signaling (40). In ventilator induced lung injury, capsaicin pretreatment restores ATP levels and antioxidant defenses, improves mitochondrial ultrastructure, and suppresses ferroptosis, thereby ameliorating lung injury *in vivo* (41). In neurodegeneration related models, including scopolamine and 3 nitropropionic acid induced injury, capsaicin restores the activity of mitochondrial complexes I–IV and improves mitochondrial permeability transition. It also normalizes antioxidant and inflammatory responses, supporting protection of neuronal mitochondrial bioenergetics and redox homeostasis (31, 42). Complementing these findings, capsaicinoid derivatives further activate AMPK dependent Nrf2 signaling in hepatocytes, inducing antioxidant gene expression and limiting lipid induced mitochondrial injury (12).

Mitochondrial quality control represents an additional regulatory layer targeted by capsaicin. The endoplasmic reticulum (ER)–mitochondria contact sites, known as mitochondria-associated membranes (MAMs), are critical for coordinating Ca^2+^ signaling, lipid metabolism, and mitochondrial dynamics, and both excessive and insufficient MAM formation can be pathological depending on disease context. In TRPV1 expressing sensory neurons and PC12 cells, repeated or high dose capsaicin exposure induces transient mitochondrial damage while simultaneously activating mitophagy and upregulating mitochondrial proteins such as COX IV, Mic60/Mitofilin, and VDAC1, suggesting a coordinated response in which damaged mitochondria are removed and replaced by a healthier organelle pool (38). In diabetic nephropathy, TRPV1 activation by dietary capsaicin attenuates pathological MAM over-formation in podocytes; TRPV1-mediated Ca^2+^ influx activates AMPK, downregulates the MAM-tethering protein FUNDC1, normalizes mitochondrial Ca^2+^ handling, and preserves mitochondrial structure and function (43). In contrast, in chronic cerebral hypoperfusion, capsaicin restores disrupted MAM integrity and MFN2 expression in hippocampal neurons, thereby improving ER–mitochondria coupling, mitochondrial bioenergetics, and cognitive performance (44). In addition, capsaicin upregulates PTEN-induced kinase 1 (PINK1) and Parkin expression, thereby enhancing mitophagy and reducing hepatic lipid accumulation (45).

Collectively, capsaicin increases mitochondrial quantity through TRPV1/AMPK/SIRT1/PGC-1α mediated biogenesis and preserves mitochondrial quality by maintaining respiratory chain function, limiting oxidative stress, enhancing antioxidant defenses, and promoting mitophagy. Importantly, these effects reflect a normalization of mitochondrial biogenesis and quality control under conditions of metabolic stress, providing a bioenergetic basis for the multitarget metabolic actions of capsaicin in CMS.

### 1.2 Molecular mechanisms of capsaicin in ameliorating insulin resistance

#### 1.2.1 Activation of the insulin signaling pathway

Capsaicin improves insulin resistance primarily by enhancing insulin receptor associated signaling. Accumulating evidence indicates that capsaicin increases insulin receptor (InsR) expression and enhances its responsiveness, thereby facilitating downstream signal transduction (46, 47). Upon InsR activation, phosphorylation of IRS proteins is increased, leading to activation of the PI3K/Akt pathway and subsequent translocation of glucose transporter type 4 (GLUT4) to the plasma membrane, ultimately promoting cellular glucose uptake and utilization (46, 48).

Mechanistically, capsaicin activates the TRPV1 channel, inducing intracellular Ca^2+^ influx that enhances IRS phosphorylation and amplifies PI3K/Akt signaling activity, thereby improving insulin sensitivity and glucose handling (47, 49, 50). In addition to its effects on insulin signaling in peripheral tissues, TRPV1 activation by capsaicin upregulates pancreatic and duodenal homeobox 1 (PDX-1), a transcription factor essential for pancreatic β cell function and insulin synthesis. This regulation involves coordinated signaling through TRPV1/PDX-1/GLUT2/glucokinase (GK), as well as TRPV1/PDX-1/IRS1/2. These effects support insulin secretion and β cell responsiveness (51). Importantly, these insulin sensitizing effects are dependent on functional TRPV1 signaling, as capsaicin fails to improve diet-induced insulin resistance in TRPV1-deficient mice (52).

#### 1.2.2 Suppression of inflammatory mediators to mitigate insulin resistance

Chronic low-grade inflammation plays a central role in the development of insulin resistance. Inflammatory signaling disrupts insulin action in adipocytes and hepatocytes by impairing key components such as IRS-1, the insulin receptor, and peroxisome proliferator activated receptor gamma (PPARγ), thereby exacerbating metabolic dysfunction (53). Capsaicin alleviates insulin resistance in part by suppressing inflammation driven interference with insulin signaling (33, 47).

A primary target of capsaicin is the Toll like receptor 4 (TLR4)/ NF-κB signaling pathway. Capsaicin potently inhibits NF-κB activation and reduces production of pro-inflammatory cytokines such as tumor necrosis factor-α (TNFα) and interleukin-6 (IL-6) (54). These cytokines activate stress kinases including c-Jun N-terminal kinase (JNK) and inhibitor of κB kinase β (IKKβ). Activation of these kinases promotes inhibitory serine phosphorylation of insulin receptor substrate 1 and weakens insulin signaling (55). IKKβ, the catalytic subunit essential for canonical NF-κB activation, directly interferes with insulin signaling by functioning as a serine kinase that phosphorylates IRS-1 on inhibitory sites (56–62). By suppressing NF-κB/IKKβ signaling, capsaicin reduces inflammation induced insulin resistance at the molecular level.

Importantly, TRPV1 activation by capsaicin exerts context dependent immunomodulatory effects, particularly under inflammatory conditions. In lipopolysaccharide primed macrophages, capsaicin induced TRPV1 activation and Ca^2+^ influx promote polarization toward an M2 macrophage phenotype characterized by anti-inflammatory properties, thereby improving the adipose tissue inflammatory microenvironment and attenuating insulin resistance (63). Capsaicin further enhances macrophage cholesterol metabolism through coordinated activation of TRPV1 and PPARγ (64). Dietary capsaicin has also been shown to lower fasting triglyceride levels via direct activation of nuclear receptors such as PPARγ, which negatively regulates NF-κB signaling (65). Together, these findings indicate that attenuation of chronic inflammation represents a key mechanism by which capsaicin mitigates insulin resistance.

#### 1.2.3 Modulation of adipose tissue function to improve insulin sensitivity

Capsaicin further enhances insulin sensitivity through functional remodeling of adipose tissue. This effect is largely mediated by activation of AMPK, a central regulator of cellular energy homeostasis and insulin responsiveness. In models of diet induced insulin resistance, capsaicin increases AMPK activity in hepatic and skeletal muscle tissues (66). As a master metabolic sensor, AMPK activation promotes fatty acid oxidation while suppressing lipogenesis and gluconeogenesis, thereby restoring energy balance and indirectly improving insulin sensitivity (14, 28, 47, 66, 67).

In adipose tissue, capsaicin induces browning of white adipose depots via TRPV1 activation, promoting the differentiation of energy dissipating beige adipocytes. This process increases systemic energy expenditure and reduces ectopic lipid deposition in both adipose tissue and liver (68). Single cell transcriptomic analyses indicate that capsaicin primarily activates TRPV1 expressing sensory nerve terminals within adipose tissue, indirectly modulating adipocyte metabolism through neuroendocrine or paracrine mechanisms rather than direct adipocyte engagement (69). These observations provide a mechanistic basis for capsaicin-induced reductions in adiposity and improvements in lipid metabolism observed in experimental models (52, 68). Capsaicin also reduces visceral fat accumulation and improves systemic lipid profiles by modulating adipokine secretion. In particular, capsaicin stimulates adiponectin release from adipose tissue (66). As a key insulin sensitizing hormone, adiponectin activates both AMPK and PPARα pathways to enhance fatty acid oxidation and glucose uptake in liver and skeletal muscle, thereby improving whole body glucolipid metabolic homeostasis (70).

The metabolic efficacy of capsaicin shows marked interindividual variability and combinatorial complexity. Sex specific differences have been reported, with meta analyses showing stronger triglyceride lowering effects in females (9). In addition, capsaicin interacts with other phytochemicals in a context dependent manner. It synergizes with berberine and catechins to suppress adipogenesis and lipid accumulation (71), whereas co administration with hesperidin attenuates its metabolic benefits (72). Importantly, capsaicin exerts tissue specific metabolic effects, preferentially enhancing hepatic fatty acid oxidation, skeletal muscle glucose uptake, and adipose tissue browning and thermogenesis (69).

Collectively, insulin resistance represents a central pathophysiological nexus in CMS, linking dysregulated glucose and lipid metabolism, chronic inflammation, and endothelial dysfunction (73, 74). Therapeutic strategies targeting insulin resistance therefore hold substantial clinical relevance (75).

### 1.3 Anti-inflammatory effects of capsaicin and its significance in cardiac metabolic syndrome

#### 1.3.1 Inhibition of inflammatory signaling pathways

Capsaicin exerts broad anti-inflammatory effects by targeting central inflammatory signaling pathways, most notably NF-κB and mitogen-activated protein kinase (MAPK), which regulate transcription of pro inflammatory mediators across multiple tissues (76–78). In lipopolysaccharide (LPS) stimulated macrophages, capsaicin suppresses activation of NF-κB and MAPK pathways, as shown by reduced phosphorylation of p38, p65, and extracellular signal regulated kinase (ERK), leading to decreased production of nitric oxide (NO), TNF-α, IL-6, and cyclooxygenase-2 (COX-2) (78, 79). In intestinal inflammation models, capsaicin attenuates inflammatory mediator synthesis and improves mucosal barrier integrity by inhibiting the TLR4/NF-κB axis in intestinal epithelial cells (77, 80).

In vascular endothelium cell, capsaicin suppresses endothelial activation by inhibiting NF-κB phosphorylation and reducing expression of vascular cell adhesion molecule-1 (VCAM-1) and intercellular adhesion molecule-1 (ICAM-1). This effect limits leukocyte adhesion and attenuates vascular inflammation (81). Capsaicin also mitigates chronic low-grade inflammation through epigenetic regulation, indirectly suppressing NF-κB activity by downregulating pro-inflammatory microRNAs such as miR-21 and miR-223 (82). These multi organ anti-inflammatory contribute to reduced endothelial injury and a lower chronic inflammatory burden in CMS.

The relevance of these effects is supported by evidence from animal models of cardiometabolic disease. In atherosclerosis, dietary capsaicin reduces systemic inflammation and delays plaque progression by inhibiting foam cell formation and leukocyte adhesion within the vascular wall (64). In salt sensitive hypertension, capsaicin exerts central anti-inflammatory effects by modulating the AMPK/Akt/Nrf2 pathway in the hypothalamic paraventricular nucleus, contributing to blood pressure stabilization (33). In models of drug induced cardiotoxicity, capsaicin pretreatment limits myocardial inflammation and reactive oxygen species (ROS) generation, conferring cardioprotection (83). Notably, genetic deletion of TRPV1 worsens outcomes after myocardial infarction, including increased mortality, cardiac inflammation, fibrosis, and functional decline, highlighting the endogenous cardioprotective role of TRPV1 in inflammatory cardiac injury (84).

#### 1.3.2 Regulation of immune cell function

Beyond suppressing inflammatory mediators, capsaicin modulates the inflammatory microenvironment by regulating immune cell function. A key mechanism involves control of macrophage polarization. Pro-inflammatory M1 macrophages secrete cytokines such as TNF-α, IL-1β, and IL-6, which sustain chronic inflammation, whereas M2 macrophages promote tissue repair and immune homeostasis. Capsaicin promotes a shift from M1 toward M2 macrophages through TRPV1 activation, thereby facilitating resolution of inflammation and restoration of metabolic homeostasis (85–87). Capsaicin also influences immune cell recruitment and activation. In intestinal epithelial cells, capsaicin inhibits PI3K/Akt dependent production of the chemokine C–C motif chemokine ligand 2 (CCL2), which reduces macrophage infiltration and attenuating multi organ inflammation associated with metabolic syndrome (88). In parallel, TRPV1 activation limits pathological recruitment of T cells and macrophages to metabolic and vascular tissues and suppresses their secretion of pro inflammatory cytokines, leading to improvements in endothelial function and systemic metabolic parameters (45, 89). Importantly, the immunomodulatory effects of capsaicin are context dependent. Under specific conditions, capsaicin stimulates leukotriene B4 receptor signaling and promotes leukotriene B4 release in keratinocyte and monocytic cell lines, resulting in transient enhancement of inflammatory responses (90).

Growing evidence further implicates capsaicin in neuro immune crosstalk and immunometabolic reprogramming. Activation of TRPV1 expressing nociceptor neurons by capsaicin induces the release of neuropeptides, including substance P (SP) and calcitonin gene–related peptide (CGRP) (91, 92), which interact with receptors on immune cells such as T lymphocytes, macrophages, neutrophils, and mast cells to modulate immune recruitment and function (93–95). Activation of TRPV1 positive vagal sensory fibers may also attenuate peripheral to central transmission of immune inflammatory signals, providing a neural basis for the systemic anti-inflammatory and cardioprotective effects of capsaicin (96).

However, TRPV1 signaling does not fully account for all immunometabolic effects of capsaicin. Independently of TRPV1, capsaicin directly inhibits the pyruvate kinase M2/lactate dehydrogenase A (PKM2/LDHA) axis in macrophages, suppressing the Warburg effect, reducing lactate accumulation, and dampening the glycolysis-driven pro-inflammatory phenotype, highlighting its dual immunometabolic regulatory capacity (97).

Clinical observations further support the relevance of this pathway to cardiometabolic pathology. In patients with concomitant coronary artery disease and type 2 diabetes, elevated TRPV1 expression in peripheral blood mononuclear cells correlates with increased circulating pro-inflammatory cytokines, including TNF-α, IL-6, and monocyte chemoattractant protein-1 (MCP-1), and independently predicts future major adverse cardiovascular events (98). These findings directly implicate TRPV1 associated inflammatory signaling in disease progression and clinical outcomes.

#### 1.3.3 Reduction of oxidative stress response

Oxidative stress is a major driver of inflammation, and the antioxidant actions of capsaicin contribute substantially to its anti-inflammatory effects. Capsaicin enhances endogenous antioxidant defenses while reducing intracellular accumulation of ROS (99, 100). Mechanistically, capsaicin disrupts the Kelch like ECH associated protein 1 (Keap1)/Nrf2 interaction, promoting nuclear translocation of Nrf2 and transcriptional activation of antioxidant enzymes, including superoxide dismutase (SOD), glutathione peroxidase (GPx), and catalase (CAT) (101, 102). Concurrently, capsaicin suppresses upstream ROS production by inhibiting the C–C chemokine receptor type 4 (CCR4)/Src/p47phox signaling cascade, thereby limiting nicotinamide adenine dinucleotide phosphate (NADPH) oxidase activity (103).

Translational evidence supports these mechanisms. In patients with metabolic syndrome, capsaicin intake increases serum SOD levels while reducing malondialdehyde (MDA), indicating restoration of systemic redox balance (104). Oxidative stress and inflammation are tightly coupled in a feed-forward loop, whereby ROS activate inflammatory pathways such as NF-κB, and inflammatory cells generate additional ROS, perpetuating tissue injury. Through TRPV1/Nrf2 signaling, capsaicin simultaneously reduces ROS accumulation and suppresses ROS driven inflammatory signaling, including NF-κB activation (103, 105). This dual action has been validated in disease models; for example, in salt-sensitive hypertension, capsaicin modulates the AMPK/Akt/Nrf2 pathway to coordinately reduce oxidative stress and inflammatory cytokine expression, conferring organ protection (33, 106). Similarly, in type 2 diabetes complicated by myocardial infarction, dietary capsaicin activates TRPV1 to attenuate oxidative stress and limit cardiac injury (107). Capsaicin has also been shown to upregulate uncoupling protein 2 (UCP2) via TRPV1-dependent mechanisms, improving endothelial function and counteracting oxidative damage in hypertensive state (108, 109).

Importantly, the biological effects of capsaicin show clear dose and tissue specificity. Excessive TRPV1 activation at high concentrations can induce Ca^2+^ overload in macrophages, leading to mitochondrial dysfunction, secondary ROS generation, and aberrant activation of the NLRP3 inflammasome, thereby promoting inflammation (110, 111). These findings underscore the necessity of defining an appropriate therapeutic window and accounting for tissue specific TRPV1 expression when considering capsaicin-based interventions. Overall, capsaicin-activated TRP channels play a central role in cardiometabolic disease by coordinately regulating oxidative stress and inflammatory pathways, providing a strong mechanistic foundation for targeting this axis in disease prevention and treatment (112).

### 1.4 Capsaicin regulation of lipid metabolism and its cardiovascular protective effects

#### 1.4.1 Inhibition of fatty acid synthesis and promotion of lipolysis by capsaicin

Capsaicin exerts robust regulatory effects on lipid metabolism by coordinately suppressing fatty acid synthesis and enhancing lipolytic pathways, processes that are critical for limiting lipid accumulation and cardiovascular risk in CMS (113). One principal mechanism involves downregulation of key lipogenic enzymes, including fatty acid synthase (FAS) and ACC, which catalyze palmitate synthesis and the committed step of fatty acid biosynthesis, respectively. Experimental studies demonstrate that capsaicin significantly reduces FAS and ACC expression at both mRNA and protein levels in cardiac tissue and liver of high-fat-diet–fed rodents, accompanied by upregulation of CPT1, a rate-limiting enzyme for mitochondrial β-oxidation (114). Consistent with these findings, phytoformulations containing capsaicin reduce cardiac lipid deposition and improve circulating lipid profiles, including total cholesterol, triglycerides, and free fatty acids, in obese rodent models (115–117). These effects are mediated, at least in part, by suppression of SREBP-1, a master transcriptional regulator of lipogenic gene expression. Capsaicin attenuates SREBP-1 signaling, thereby repressing downstream adipogenic pathways and reinforcing inhibition of fatty acid synthesis (11, 117, 118).

In parallel, capsaicin promotes lipid mobilization by enhancing lipolysis. Capsaicin and its analogs increase the expression and activity of lipolytic enzymes, including hormone-sensitive lipase (HSL), adipose triglyceride lipase (ATGL), lysosomal acid lipase, and lipoprotein lipase. For example, the capsaicin analog nonivamide upregulates HSL and ATGL in porcine subcutaneous adipocytes, stimulating lipolysis and inducing a browning-associated transcriptional program (119). In obese rat models, capsaicin-based formulations similarly increase lipase expression in cardiac tissue, correlating with reduced lipid droplet accumulation and improved cardiac performance (117). This coordinated suppression of lipogenesis and enhancement of lipolysis is essential for maintaining lipid homeostasis and preventing ectopic lipid deposition, a major driver of cardiovascular dysfunction.

Beyond direct enzymatic regulation, capsaicin modulates lipid metabolism through bile acid signaling. Capsaicin increases circulating bile acids such as chenodeoxycholic acid and deoxycholic acid, which activate the farnesoid X receptor (FXR) pathway and contribute to reductions in triglyceride and cholesterol levels (120, 121). At the cellular level, capsaicin downregulates lipogenic genes including SREBP-1c, FAS, and ACC, while upregulating lipid oxidation–related genes such as PPARα and PPARδ in hepatocytes (12, 45).

At the cellular level, capsaicin’s activation of TRPV1 channels plays a pivotal role in these metabolic effects. TRPV1 activation inhibits the dimerization of PKM2 and reduces the activation of SREBP1, thereby suppressing lipogenesis in microglia and potentially other tissues (97, 122). Moreover, TRPV1 activation can restore mitochondrial membrane potential, reduce mitochondrial ROS, alleviate oxidative stress and mitochondrial dysfunction, and thus indirectly ameliorate lipid metabolism disorders.

#### 1.4.2 Regulation of cholesterol metabolism

Capsaicin also exerts pronounced regulatory effects on cholesterol metabolism, contributing to its cardiovascular protective profile through coordinated modulation of cholesterol transport, efflux, and catabolism. In apolipoprotein E–deficient mice, capsaicin reduces atherosclerotic lesion formation by limiting cholesterol influx via scavenger receptor A (SR-A) and enhancing cholesterol efflux through upregulation of ATP-binding cassette transporter A1 (ABCA1) in macrophages. This shift reduces oxidized low-density lipoprotein uptake, inhibits foam cell formation, and is mediated predominantly through TRPV1 and PPARγ signaling (64, 123). TRPV1 activation further promotes liver X receptor alpha (LXRα)–dependent induction of ABCA1 and ATP-binding cassette transporter G1 (ABCG1), facilitating cholesterol efflux and suppressing lipid accumulation (124).

Furthermore, capsaicin alters bile acid composition by promoting the conversion of cholesterol into bile acids via increased hepatic expression of cholesterol 7α-hydroxylase (CYP7A1), the rate limiting enzyme in bile acid synthesis. Notably, this effect was observed even in germ free mice lacking intestinal flora and TRPV1 channels, indicating that capsaicin’s modulation of cholesterol metabolism can occur independently of both gut microbiota and TRPV1 signaling. By changing bile acid profiles, capsaicin inhibits fibroblast growth factor 15 (Fgf15) expression in the colon, which normally suppresses CYP7A1, thereby enhancing cholesterol catabolism and reducing plasma total cholesterol and triglyceride levels (125).

Clinical evidence supports these experimental observations. Systematic reviews and meta-analyses demonstrate that capsaicin supplementation significantly lowers total cholesterol and low-density lipoprotein cholesterol levels in individuals with metabolic syndrome or overweight/obesity (9, 126). At the cellular level, capsaicin activates the LKB1/AMPK axis in hepatocytes, promoting metabolic reprogramming. Capsaicin also upregulates PINK1 and Parkin, initiating mitophagy and improving mitochondrial quality control, thereby reducing hepatic lipid accumulation (45).

In addition, capsaicin influences cholesterol metabolism through modulation of gut microbiota composition. In rats fed a high fat diet, capsaicin, especially when combined with dietary fibers, increases the abundance of beneficial bacteria such as *Akkermansia* and *Allobaculum*. These microbial changes are associated with improved lipid profiles and reduced LDL-C levels (120, 127). Remodeling of the gut microbiome through enhanced short-chain fatty acid production, altered bile acid metabolism, and suppression of inflammatory signaling forms a synergistic mechanism that improves cholesterol handling and reduces cardiovascular risk. These findings provide experimental support for the use of capsaicin or its derivatives as functional food components or nutritional interventions.

Beyond metabolic regulation, capsaicin and its analogs may exert direct molecular interactions influencing cholesterol homeostasis. For instance, homocapsaicin II induces ferroptosis in colorectal cancer cells through a cholesterol centrosome amplification axis, highlighting a novel link between cholesterol metabolism and cellular death pathways (128). Although this mechanism is cancer specific, it underscores the broader impact of capsaicin-related compounds on cholesterol dynamics.

#### 1.4.3 Protection of vascular function and remodeling

Capsaicin, the active component of chili peppers, exerts multifaceted protective effects on vascular endothelial function, which is crucial for mitigating cardiovascular diseases and metabolic syndrome. Acute capsaicin treatment significantly increases endothelial p-eNOS levels in wild-type mice. The mechanism involves capsaicin acting on endothelial TRPV1, causing Ca^2+^ influx, which subsequently promotes eNOS phosphorylation and increases NO, BH₄, and cyclic guanosine monophosphate (cGMP) levels, enhancing vasodilation. These effects can be inhibited by Ca^2+^ chelators or TRPV1 antagonists (129). Additionally, capsaicin also induces vasodilation in various blood vessels (mesenteric, carotid, coronary) through both endothelium-NO-dependent pathways and endothelium-independent mechanisms involving potassium channel activation and Ca^2+^ influx regulation (130, 131). Experimental research in rat aortic endothelial cells showed that capsaicin-induced TRPV1 activation elevates NO levels by up to 29%, alongside increased levels of the essential cofactor BH4 and cGMP, which are critical for eNOS function and downstream signaling, respectively. This cascade culminates in vasodilation and improved endothelial function (132).

However, evidence from TRPV1 and TRPV4 knockout mouse models indicates that capsaicin-induced vasorelaxation does not rely exclusively on TRPV1 signaling. In these models, vasorelaxation occurred predominantly through TRPV4-mediated endothelium-dependent hyperpolarization, with only a marginal contribution from the TRPV1, NO, and PGI2 pathway. Mechanistically, capsaicin activates endothelial TRPV4 and intermediate-conductance Ca^2+^-activated potassium channels, which induce membrane hyperpolarization and promote vasorelaxation (133).

In addition to promoting vasodilation, under high sodium culture conditions or high salt stimulation, capsaicin significantly inhibited the proliferation of rat aortic smooth muscle cells in a dose dependent manner, accompanied by upregulated TRPV1 expression (134). TRPV1 is expressed in both rat and human pulmonary artery smooth muscle cells, and pharmacological blockade of TRPV1 suppresses hypoxia-induced Ca^2+^ entry and cell proliferation. In contrast, in a rat hindlimb ischemia model, TRPV1 activation enhanced endothelial cell proliferation via Ca^2+^ dependent transcription factors, including NFAT-1, calsenilin, and MEF2C, thereby promoting positive remodeling of collateral vessels. This suggests a balanced regulation of vascular cell proliferation that favors endothelial repair while restraining VSMC overgrowth (135). This dual regulatory role is essential for preventing maladaptive vascular remodeling, which contributes to hypertension and other cardiovascular pathologies.

Moreover, after TRPV1 activation, the total antioxidant capacity (TAC) in rat aortic tissue significantly increased, highlighting the antioxidant effects of capsaicin (132). Capsaicin also modulates neurovascular interactions, where sensory neuron-derived peptides such as CGRP stimulate endothelial NO production, further supporting vascular health and resilience (136). Clinically, topical application of capsaicin in men can enhance post-exercise microvascular reperfusion response and improve local blood flow recovery. Women did not show significant blood pressure changes at the same dose, but enhanced microvascular responsiveness was still observed in younger subjects. No significant effect was seen in elderly groups, suggesting the existence of sex and age differences and indicating the need for personalized approaches in therapeutic applications (137).

Beyond regulating vascular tone, endothelial dysfunction is an early driver of atherogenesis and plaque progression in cardiometabolic syndrome. In this context, capsaicin-mediated enhancement of endothelial NO bioavailability, together with its antioxidant and anti-inflammatory actions, may help preserve endothelial homeostasis and thereby attenuate pro-atherogenic processes.

### 1.5 The capsaicin–gut microbiota axis in cardiometabolic regulation

#### 1.5.1 Mechanisms by which capsaicin regulates gut microbiota composition

Capsaicin modulates gut microbiota composition through coordinated effects on the intestinal epithelial barrier, luminal microenvironment, and host–microbe interactions. At the epithelial level, capsaicin enhances the integrity of the mucus layer and upregulates tight junction proteins, including zonula occludens-1 (ZO-1) and occludin, thereby strengthening barrier function and shaping the microbial niche within the intestinal lumen (138, 139). By improving epithelial integrity and mucosal immunity, capsaicin indirectly restricts colonization by pathogenic bacteria while favoring beneficial commensals.

Capsaicin also alters luminal factors such as pH and bile acid composition, creating selective ecological pressures that promote the growth of beneficial taxa, including *Akkermansia muciniphila* and Bifidobacterium, while reducing the Firmicutes/Bacteroidetes ratio and suppressing potentially pathogenic families such as Enterobacteriaceae (140–144). In parallel, capsaicin induces the expression of antimicrobial peptides, such as regenerating islet-derived protein 3 gamma (Reg3γ), and modulates bile acid metabolism, both of which contribute to maintaining microbial diversity and community stability (120, 143, 145).

Consistent with these mechanisms, capsaicin supplementation in animal models increases the abundance of short-chain fatty acid (SCFA)–producing bacteria, including *Allobaculum* and *Faecalibaculum*, while reducing pro-inflammatory taxa, resulting in improved metabolic phenotypes (146, 147). Human *in vitro* fecal fermentation studies further corroborate these findings, demonstrating increased butanoic acid production and enhanced microbial diversity following capsaicin exposure (146, 148). Importantly, excessive doses or prolonged capsaicin intake may disrupt barrier integrity and microbial balance, emphasizing the importance of dose, duration, and host context in microbiota-mediated effects (149–151).

#### 1.5.2 Metabolic regulatory pathways mediated by gut microbiota

Capsaicin induced alterations in gut microbiota composition translate into downstream metabolic benefits through multiple microbial signaling pathways. A prominent mechanism involves increased production of SCFAs, including acetate, propionate, and butyrate, which contribute to improved glucose homeostasis and attenuation of dyslipidemia (142, 146, 151–153). These SCFAs activate G-protein-coupled receptors 41 and 43 (GPR41 and GPR43) and inhibit histone deacetylases (HDACs), thereby enhancing lipid oxidation, insulin sensitivity, and energy expenditure (141, 142, 154).

In addition, microbiota remodeling induced by capsaicin modulates bile acid metabolism by reshaping the composition and pool size of primary and secondary bile acids (120, 140). These bile acids act as signaling molecules that activate the FXR and the G protein-coupled bile acid receptor 1 (TGR5), pathways known to regulate lipid metabolism, promote cholesterol efflux, increase energy expenditure, and reduce hepatic steatosis and atherosclerotic risk (140, 145, 155, 156). The gut microbiota further contributes to metabolic regulation through modulation of tryptophan metabolism, generating bioactive metabolites that influence systemic inflammation and metabolic homeostasis (147, 157).

Through these interconnected pathways, capsaicin induced shifts in microbiota composition function as an upstream amplifier of host metabolic signaling, reinforcing improvements in lipid handling, insulin sensitivity, and cardiovascular risk profiles.

#### 1.5.3 Integrated effects of capsaicin-gut microbiota interaction on cardiometabolic syndrome

The interaction between capsaicin and gut microbiota exerts integrated protective effects on CMS by simultaneously improving barrier integrity, suppressing systemic inflammation, and modulating immune responses. By upregulating tight junction proteins and enhancing mucus secretion, capsaicin reduces translocation of endotoxins such as LPS into the circulation, thereby alleviating metabolic endotoxemia (138, 139, 152). In mouse models fed a high fat diet, capsaicin lowers circulating LPS levels, inhibits TLR4 signaling, and suppresses NF-κB activation, as reflected by reduced phosphorylation of p65 and inhibitor of κB alpha (IκBα). These changes reduce pro-inflammatory cytokine production, including TNF-α, IL-1β, and IL-6, and help alleviate chronic low-grade inflammation (141, 158).

Beyond innate immune signaling, interactions between capsaicin and the gut microbiota influence macrophage polarization and cytokine balance, favoring anti-inflammatory phenotypes and reducing adipose tissue inflammation (139, 140). Hence, this interaction is best conceptualized as a bidirectional, positive feedback cycle. Capsaicin appears to enhance intestinal barrier integrity and host immune tone while concurrently influencing the composition and function of the gut microbiota. In turn, the adapted microbiota generates anti-inflammatory metabolites, such as SCFAs, which further stabilize the gut environment and reinforce a health-promoting microbial ecosystem. Within this positive-feedback framework, microbiota–immune interactions act in concert with the antioxidant properties of capsaicin to mitigate oxidative stress, preserve endothelial function, and reduce the risk of hypertension and atherosclerosis (105, 158). Collectively, these effects contribute to improved insulin sensitivity, lipid profiles, and vascular homeostasis, ultimately reducing the burden of obesity-associated cardiometabolic complications. Importantly, the magnitude of these benefits depends on capsaicin dose, exposure duration, and the host’s baseline microbiota composition.

#### 1.5.4 Preclinical and clinical research progress

Preclinical studies in models of diet induced obesity and atherosclerosis consistently show that capsaicin supplementation remodels gut microbiota composition, increasing beneficial taxa such as Akkermansia and Bifidobacterium while suppressing pathogenic bacteria. These microbiota changes are accompanied by improvements in body weight, serum lipid profiles, glucose tolerance, and inflammatory markers (140, 142, 159). In apolipoprotein E deficient mice, capsaicin reduces atherosclerotic plaque formation through microbiota-dependent mechanisms involving bile acid metabolism and FXR signaling (140). In diabetic rodent models, capsaicin enhances the hypoglycemic efficacy of metformin and restores intestinal barrier function in parallel with favorable microbiota shifts (160).

Human data remain limited but supportive. *In vitro* fecal fermentation studies show that capsaicin increases SCFA production and microbial diversity (146). However, adverse effects have been reported at high doses or with prolonged intake, including barrier disruption and exacerbation of anxiety-like behaviors in diabetic models, underscoring the importance of dose optimization and individual variability (139, 149). Key research gaps include defining interindividual microbiota responsiveness, long-term safety, and translational relevance in humans. Future studies should prioritize well-controlled clinical trials, personalized nutrition strategies, and mechanistic investigations to fully harness the therapeutic potential of the capsaicin–gut microbiota axis in CMS.

## 2 Conclusion

**Figure 2:**
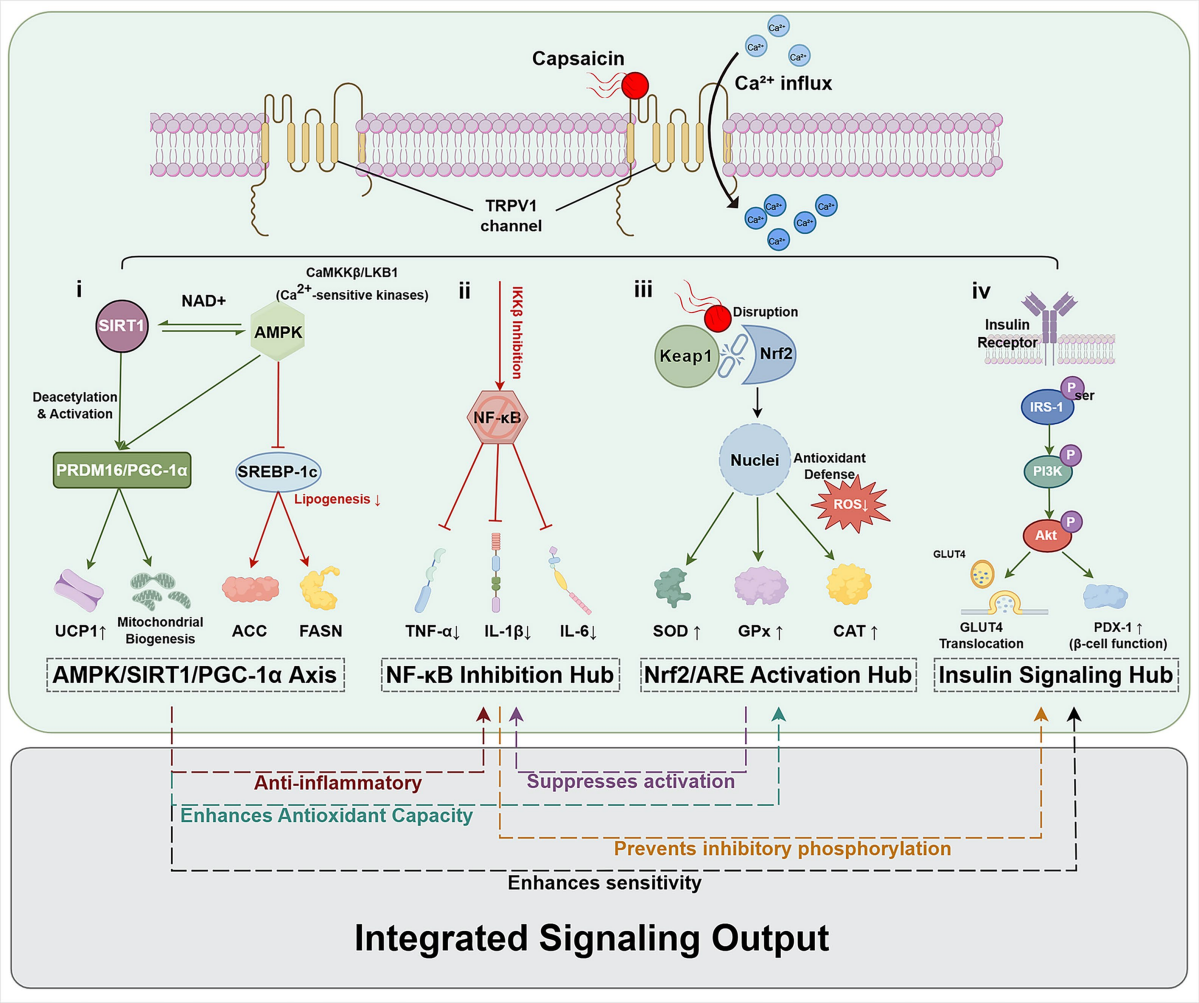
TRPV1-centered signaling networks through which capsaicin exerts pleiotropic biological effects. Dietary capsaicin binds to and activates TRPV1 channels on the cell membranes, leading to Ca^2+^ influx and engagement of multiple interconnected signaling modules: (i) metabolic regulation hub (AMPK/SIRT1/PGC-1α axis): Ca^2+^-sensitive kinases such as CaMKKβ/LKB1 activate AMPK, which in turn increases SIRT1 activity and upregulates the PRDM16/PGC-1α program. This axis promotes UCP1 expression and mitochondrial biogenesis, while AMPK concomitantly inhibits lipogenesis by suppressing SREBP-1c and its downstream enzymes ACC and FASN. (ii) Inflammatory signaling hub (NF-κB inhibition): capsaicin dampens IKKβ activity and NF-κB activation, thereby reducing the production of pro-inflammatory cytokines TNF-α, IL-1β, and IL-6. (iii) Antioxidant defense hub (Nrf2/ARE activation): disruption of the Keap1/Nrf2 complex allows Nrf2 nuclear translocation and transcriptional activation via antioxidant response element (ARE), leading to increased expression of antioxidant enzymes such as SOD, GPx, and CAT, limiting ROS accumulation. (iv) Insulin signaling hub: capsaicin improves insulin signaling by preserving insulin-receptor–IRS-1–PI3K–Akt signaling, facilitating GLUT4 translocation in insulin-sensitive tissues and supporting β-cell function via PDX-1. The integrated outcome of these pathways, summarized at the bottom, is a coordinated anti-inflammatory and antioxidant response, prevention of inhibitory phosphorylation within the insulin pathway, and enhanced insulin sensitivity, collectively contributing to improved metabolic and cardiometabolic homeostasis.

Capsaicin emerges as a promising multitarget bioactive compound capable of modulating several core pathological processes underlying CMS. Through activation of the TRPV1 channel and downstream AMPK signaling, capsaicin enhances mitochondrial function, increases energy expenditure, and improves metabolic flexibility, thereby addressing fundamental disturbances in energy homeostasis. These major metabolic effects are further enhanced through multiple synergistic interactions such as improving insulin signaling, inhibiting chronic low-grade inflammation, remodeling of adipose tissue function, and reducing oxidative stress. (Figure 2, Table 1).

**Table 1:** Multitarget mechanisms by which capsaicin modulates cardiometabolic syndrome related pathophysiological processes.

Mechanism of action	Key signaling pathways or targets	Affected CMS pathophysiological processes	Level of experimental evidence	References
Activation of TRPV1	Ca^2+^ influx, sympathetic activation	↑ Energy expenditure, ↑ thermogenesis	Cellular, animal, clinical	(5–7, 19–23)
Activation of AMPK	TRPV1-Ca^2+^ signaling; CaMKK2 (CaMKKβ)/LKB1	↑ Insulin sensitivity, ↑ lipid metabolic homeostasis	Cellular, animal	(10–12, 14, 28–31, 47, 66, 67)
Inhibition of NF-κB	Suppression of IKKβ/p65 phosphorylation	↓ Chronic low-grade inflammation	Cellular, animal	(54, 56–62, 76–81)
Activation of Nrf2	Keap1/Nrf2 dissociation	↓ Oxidative stress	Cellular, animal	(33, 101–103, 105–107)
Activation of endothelial TRPV1-eNOS signaling	TRPV1-Ca^2+^ influx; eNOS phosphorylation; NO/BH₄/cGMP signaling	↑ Endothelial function, ↑ vasodilation, ↓ endothelial dysfunction and atherogenic activation	Cellular, animal	(129–132)
Modulation of gut microbiota	SCFAs, bile acid signaling	↓ Metabolic endotoxemia, ↓ systemic inflammation	Animal, ex vivo human	(120, 138–142, 145–148, 155, 156, 159, 160)

Beyond intracellular metabolic regulation, capsaicin favorably influences lipid homeostasis by inhibiting lipogenesis, promoting fatty acid oxidation, and improving cholesterol handling, collectively contributing to vascular protection. In addition, modulation of gut microbiota composition and reinforcement of intestinal barrier integrity provide an extra layer of systemic metabolic control, underscoring the importance of the gut–metabolism axis in cardiometabolic regulation.

Despite compelling mechanistic and preclinical evidence, clinical translation of capsaicin-based interventions remains constrained by variability in dosing, bioavailability, and interindividual responsiveness. (Table 2) To facilitate the translation of mechanistic insights into clinical application, a phased, biomarker-guided approach incorporating preclinical pharmacokinetic studies, early-phase dose-escalation trials, and subsequent long-term randomized controlled trials may help define therapeutic dose ranges, inform safety considerations, and account for interindividual variability.

**Table 2:** Cardiometabolic effects of capsaicin in clinical and experimental studies.

Study type	Population/model	Intervention	Main metabolic outcomes	References
Human studies (RCT & cross-sectional)	Individuals with obesity or metabolic syndrome; healthy adults	Capsaicin supplementation (X mg/day for Y weeks) or acute capsaicin intake	↓ Triglycerides (TG), ↓ Total cholesterol (TC), ↑ Energy expenditure, ↑ Brown adipose tissue (BAT) activity (assessed by PET/CT)	(9, 20–22)
Animal model	High fat diet fed mice	Dietary capsaicin supplementation	↓ Body weight gain, ↓ hepatic lipid accumulation	(11, 37)
In vitro study	LKB1-deficient HepG2 cells	Capsaicin treatment	↓ Lipid accumulation, ↑ AMPK phosphorylation	(10, 12)

In summary, the integrated actions of capsaicin across energy metabolism, insulin sensitivity, inflammatory and oxidative pathways, lipid regulation, vascular function, and gut microbiota modulation support its potential as an adjunctive therapeutic approach for the management of CMS. Continued interdisciplinary research will be essential to translate these mechanistic insights into clinically actionable interventions capable of improving long-term metabolic and cardiovascular outcomes.

## References

[1] Szallasi A. Dietary capsaicin: a spicy way to improve cardio-metabolic health? Biomolecules. (2022) 12. doi: 10.3390/biom12121783, 36551210 PMC9775666

[2] Ghobadian B Shahrokhi SA Talabon SR Notash V Sedgi FM Rahimlou M. Association of diet quality, dietary acid load, and dietary antioxidant index with cardiometabolic and NAFLD risk factors among patients with metabolic syndrome: a cross-sectional study. Food Sci Nutr. (2025) 13:e71102. doi: 10.1002/fsn3.71102, 41111892 PMC12534197

[3] Sveeggen TM Bagher P Toba H Lindsey ML Ritchie RH Miksztowicz VJ . Guidelines for diet-induced models of cardiometabolic syndrome. Am J Physiol Heart Circ Physiol. (2025) 329:H974–88. doi: 10.1152/ajpheart.00359.2025, 40912726 PMC12573438

[4] Liang W Lan Y Chen C Song M Xiao J Huang Q . Modulating effects of capsaicin on glucose homeostasis and the underlying mechanism. Crit Rev Food Sci Nutr. (2023) 63:3634–52. doi: 10.1080/10408398.2021.1991883, 34657531

[5] Baskaran P Krishnan V Ren J Thyagarajan B. Capsaicin induces browning of white adipose tissue and counters obesity by activating TRPV1 channel-dependent mechanisms. Br J Pharmacol. (2016) 173:2369–89. doi: 10.1111/bph.13514, 27174467 PMC4945767

[6] Baskaran P Krishnan V Fettel K Gao P Zhu Z Ren J . TRPV1 activation counters diet-induced obesity through sirtuin-1 activation and PRDM-16 deacetylation in brown adipose tissue. Int J Obes. (2017) 41:739–49. doi: 10.1038/ijo.2017.16, 28104916 PMC5413365

[7] Baskaran P Gustafson N Chavez N. TRPV1 activation antagonizes high-fat diet-induced obesity at thermoneutrality and enhances UCP-1 transcription via PRDM-16. Pharmaceuticals (Basel). (2024) 17. doi: 10.3390/ph17081098, 39204203 PMC11359803

[8] Kwon J Kim DY Cho KJ Hashimoto M Matsuoka K Kamijo T . Pathophysiology of overactive bladder and pharmacologic treatments including β3-adrenoceptor agonists -basic research perspectives. Int Neurourol J. (2024) 28:12–33. doi: 10.5213/inj.2448002.001, 38461853 PMC10932578

[9] Jiang Z Qu H Lin G Shi D Chen K Gao Z. Lipid-lowering efficacy of the capsaicin in patients with metabolic syndrome: a systematic review and meta-analysis of randomized controlled trials. Front Nutr. (2022) 9:812294. doi: 10.3389/fnut.2022.812294, 35299764 PMC8923259

[10] Bort A Sanchez BG Mateos-Gomez PA Diaz-Laviada I Rodriguez-Henche N. Capsaicin targets lipogenesis in HepG2 cells through AMPK activation, AKT inhibition and PPARs regulation. Int J Mol Sci. (2019) 20. doi: 10.3390/ijms20071660, 30987128 PMC6480012

[11] Yang H Xie J Wang N Zhou Q Lu Y Qu Z . Effects of Miao sour soup on hyperlipidemia in high-fat diet-induced obese rats via the AMPK signaling pathway. Food Sci Nutr. (2021) 9:4266–77. doi: 10.1002/fsn3.2394, 34401077 PMC8358355

[12] Elkhedir A Yahya A Jadelrab EH Salih MM Albahi A Haran Y . Capsaicinoid glucoside attenuates lipid accumulation in HepG2 cells through TRPV1/AMPK-dependent signaling pathway. Food Sci Nutr. (2025) 13:e70564. doi: 10.1002/fsn3.70564, 40612135 PMC12221993

[13] Abdillah AM Lee JY Lee YR Yun JW. Modulatory roles of capsaicin on thermogenesis in C2C12 myoblasts and the skeletal muscle of mice. Chem Biol Interact. (2025) 407:111380. doi: 10.1016/j.cbi.2025.111380, 39800145

[14] Uddandrao VVS Krishna D Vasantha MN Jayashree G Roy A Ravikkumar VR . Combination of hydroxycitric acid and capsaicin regulates IRS-1/AMPK-mTOR-SREBP-1c axis/NLRP3-NF-κB/Nrf-2-ARE signaling pathways to ameliorate the two-hit process in high-fat diet-induced hepatic steatosis. Biochim Biophys Acta Mol Cell Biol Lipids. (2025) 1870:159682. doi: 10.1016/j.bbalip.2025.159682, 40854370

[15] Engin A. Reappraisal of adipose tissue inflammation in obesity. Adv Exp Med Biol. (2024) 1460:297–327. doi: 10.1007/978-3-031-63657-8_10, 39287856

[16] Shulman GI. Ectopic fat in insulin resistance, dyslipidemia, and cardiometabolic disease. N Engl J Med. (2014) 371:1131–41. doi: 10.1056/NEJMra1011035 25229917

[17] Wang H Cai W Zeng H Xu Z Luo X Wu J . Inflammatory markers mediate the association between weight-adjusted waist circumference and mortality in patients with cardiometabolic syndrome. Sci Rep. (2025) 15:8505. doi: 10.1038/s41598-025-92733-y, 40075096 PMC11903782

[18] Wei X Guo Z Wang J Gao D Xu Q Hua S. Gut mycobiome in cardiometabolic disease progression: current evidence and future directions. Front Microbiol. (2025) 16:1659654. doi: 10.3389/fmicb.2025.1659654, 41140404 PMC12546299

[19] Takeda Y Dai P. Capsaicin directly promotes adipocyte browning in the chemical compound-induced brown adipocytes converted from human dermal fibroblasts. Sci Rep. (2022) 12:6612. doi: 10.1038/s41598-022-10644-8, 35459786 PMC9033854

[20] Yoneshiro T Aita S Kawai Y Iwanaga T Saito M. Nonpungent capsaicin analogs (capsinoids) increase energy expenditure through the activation of brown adipose tissue in humans. Am J Clin Nutr. (2012) 95:845–50. doi: 10.3945/ajcn.111.018606, 22378725

[21] Sun L Camps SG Goh HJ Govindharajulu P Schaefferkoetter JD Townsend DW . Capsinoids activate brown adipose tissue (BAT) with increased energy expenditure associated with subthreshold 18-fluorine fluorodeoxyglucose uptake in BAT-positive humans confirmed by positron emission tomography scan. Am J Clin Nutr. (2018) 107:62–70. doi: 10.1093/ajcn/nqx025, 29381803

[22] Fuse S Endo T Tanaka R Kuroiwa M Ando A Kume A . Effects of capsinoid intake on brown adipose tissue vascular density and resting energy expenditure in healthy, middle-aged adults: a randomized, double-blind, placebo-controlled study. Nutrients. (2020) 12. doi: 10.3390/nu12092676, 32887379 PMC7551765

[23] Irandoost P Lotfi Yagin N Namazi N Keshtkar A Farsi F Mesri Alamdari N . The effect of capsaicinoids or capsinoids in red pepper on thermogenesis in healthy adults: a systematic review and meta-analysis. Phytother Res. (2021) 35:1358–77. doi: 10.1002/ptr.6897, 33063385

[24] Kazak L Chouchani ET Jedrychowski MP Erickson BK Shinoda K Cohen P . A creatine-driven substrate cycle enhances energy expenditure and thermogenesis in beige fat. Cell. (2015) 163:643–55. doi: 10.1016/j.cell.2015.09.035, 26496606 PMC4656041

[25] Rahbani JF Roesler A Hussain MF Samborska B Dykstra CB Tsai L . Creatine kinase B controls futile creatine cycling in thermogenic fat. Nature. (2021) 590:480–5. doi: 10.1038/s41586-021-03221-y, 33597756 PMC8647628

[26] Ikeda K Kang Q Yoneshiro T Camporez JP Maki H Homma M . UCP1-independent signaling involving SERCA2b-mediated calcium cycling regulates beige fat thermogenesis and systemic glucose homeostasis. Nat Med. (2017) 23:1454–65. doi: 10.1038/nm.4429, 29131158 PMC5727902

[27] Guarnieri AR Benson TW Tranter M. Calcium cycling as a mediator of thermogenic metabolism in adipose tissue. Mol Pharmacol. (2022) 102:51–9. doi: 10.1124/molpharm.121.000465, 35504660 PMC9341262

[28] Bort A Sanchez BG Spinola E Mateos-Gomez PA Rodriguez-Henche N Diaz-Laviada I. The red pepper's spicy ingredient capsaicin activates AMPK in HepG2 cells through CaMKKβ. PLoS One. (2019) 14:e0211420. doi: 10.1371/journal.pone.0211420, 30695053 PMC6350977

[29] Vahidi Ferdowsi P Ahuja KDK Beckett JM Myers S. TRPV1 activation by capsaicin mediates glucose oxidation and ATP production independent of insulin signalling in mouse skeletal muscle cells. Cells. (2021) 10. doi: 10.3390/cells10061560, 34205555 PMC8234135

[30] Sanchez BG Bort A Mora-Rodriguez JM Diaz-Laviada I. The natural chemotherapeutic capsaicin activates AMPK through LKB1 kinase and TRPV1 receptors in prostate Cancer cells. Pharmaceutics. (2022) 14. doi: 10.3390/pharmaceutics14020329, 35214061 PMC8880011

[31] Zeng H Shi N Peng W Yang Q Ren J Yang H . Effects of capsaicin on glucose uptake and consumption in hepatocytes. Molecules. (2023) 28. doi: 10.3390/molecules28135258, 37446918 PMC10343879

[32] Zhu SL Wang ML He YT Guo SW Li TT Peng WJ . Capsaicin ameliorates intermittent high glucose-mediated endothelial senescence via the TRPV1/SIRT1 pathway. Phytomedicine. (2022) 100:154081. doi: 10.1016/j.phymed.2022.154081, 35405615

[33] Jia XY Yang Y Jia XT Jiang DL Fu LY Tian H . Capsaicin pretreatment attenuates salt-sensitive hypertension by alleviating AMPK/Akt/Nrf2 pathway in hypothalamic paraventricular nucleus. Front Neurosci. (2024) 18:1416522. doi: 10.3389/fnins.2024.1416522, 38872941 PMC11169651

[34] Wei J Lin J Zhang J Tang D Xiang F Cui L . TRPV1 activation mitigates hypoxic injury in mouse cardiomyocytes by inducing autophagy through the AMPK signaling pathway. Am J Physiol Cell Physiol. (2020) 318:C1018–29. doi: 10.1152/ajpcell.00161.2019, 32293932

[35] Liu X Li Z Zhao Q Zhou X Wang Y Zhao G . Capsaicin reverses cisplatin resistance in tongue squamous cell carcinoma by inhibiting the Warburg effect and facilitating mitochondrial-dependent apoptosis via the AMPK/AKT/mTOR axis. Cell Biol Int. (2024) 48:1097–110. doi: 10.1002/cbin.12169, 38706122

[36] Que T Ren B Fan Y Liu T Hou T Dan W . Capsaicin inhibits the migration, invasion and EMT of renal cancer cells by inducing AMPK/mTOR-mediated autophagy. Chem Biol Interact. (2022) 366:110043. doi: 10.1016/j.cbi.2022.110043, 36044967

[37] Baskaran P Christensen R Bruce KD Eckel RH. Obesity-induced MASLD is reversed by capsaicin via hepatic TRPV1 activation. Curr Issues Mol Biol. (2025) 47. doi: 10.3390/cimb47080618, 40864772 PMC12384517

[38] Shibata M Kayama Y Takizawa T Ibata K Shimizu T Yuzaki M . Resilience to capsaicin-induced mitochondrial damage in trigeminal ganglion neurons. Mol Pain. (2020) 16:1744806920960856. doi: 10.1177/1744806920960856, 32985330 PMC7536481

[39] Ghorbanpour A Salari S Baluchnejadmojarad T Roghani M. Capsaicin protects against septic acute liver injury by attenuation of apoptosis and mitochondrial dysfunction. Heliyon. (2023) 9:e14205. doi: 10.1016/j.heliyon.2023.e14205, 36938442 PMC10018474

[40] Qiao Y Hu T Yang B Li H Chen T Yin D . Capsaicin alleviates the deteriorative mitochondrial function by upregulating 14-3-3 η in anoxic or anoxic/reoxygenated cardiomyocytes. Oxidative Med Cell Longev. (2020) 2020:1750289. doi: 10.1155/2020/1750289, 32190168 PMC7073486

[41] Lin J Ou H Luo B Ling M Lin F Cen L . Capsaicin mitigates ventilator-induced lung injury by suppressing ferroptosis and maintaining mitochondrial redox homeostasis through SIRT3-dependent mechanisms. Mol Med. (2024) 30:148. doi: 10.1186/s10020-024-00910-y, 39266965 PMC11391744

[42] Tyagi S Thakur AK. Neuropharmacological study on capsaicin in scopolamine-injected mice. Curr Alzheimer Res. (2023) 20:660–76. doi: 10.2174/0115672050286225231230130613, 38213170

[43] Wei X Wei X Lu Z Li L Hu Y Sun F . Activation of TRPV1 channel antagonizes diabetic nephropathy through inhibiting endoplasmic reticulum-mitochondria contact in podocytes. Metabolism. (2020) 105:154182. doi: 10.1016/j.metabol.2020.154182, 32061660

[44] Ouyang M Zhang Q Shu J Wang Z Fan J Yu K . Capsaicin ameliorates the loosening of mitochondria-associated endoplasmic reticulum membranes and improves cognitive function in rats with chronic cerebral hypoperfusion. Front Cell Neurosci. (2022) 16:822702. doi: 10.3389/fncel.2022.822702, 35370565 PMC8968035

[45] Song H Xie M Xu H Liu J Su A Liu Y . Capsaicin regulated lipid metabolism in HepG2 via mitochondrial autophagy PINK1/Parkin pathway. Gene. (2025) 968:149752. doi: 10.1016/j.gene.2025.149752, 40907586

[46] Tang W Fan Y. SIRT6 as a potential target for treating insulin resistance. Life Sci. (2019) 231:116558. doi: 10.1016/j.lfs.2019.116558, 31194993

[47] Ferdowsi PV Ahuja KDK Beckett JM Myers S. Capsaicin and zinc Signalling pathways as promising targets for managing insulin resistance and type 2 diabetes. Molecules. (2023) 28. doi: 10.3390/molecules28062861, 36985831 PMC10051839

[48] Semwal DK Kumar A Aswal S Chauhan A Semwal RB. Protective and therapeutic effects of natural products against diabetes mellitus via regenerating pancreatic β-cells and restoring their dysfunction. Phytother Res. (2021) 35:1218–29. doi: 10.1002/ptr.6885, 32987447

[49] Sobrevilla-Navarro AA Ramos-Lopez O Landeros-Sánchez B Sánchez-Parada MG González-Santiago AE. Computer-aided ligand identification of capsaicinoids and their potential functions in metabolic diseases. Mol Divers. (2025). doi: 10.1007/s11030-025-11182-x, 40252144

[50] Wang P Yan Z Zhong J Chen J Ni Y Li L . Transient receptor potential vanilloid 1 activation enhances gut glucagon-like peptide-1 secretion and improves glucose homeostasis. Diabetes. (2012) 61:2155–65. doi: 10.2337/db11-1503, 22664955 PMC3402317

[51] Zhang S Tang L Xu F Hui Y Lu H Liu X. TRPV1 receptor-mediated hypoglycemic mechanism of capsaicin in Streptozotocin-induced diabetic rats. Front Nutr. (2021) 8:750355. doi: 10.3389/fnut.2021.750355, 34692753 PMC8526734

[52] Lee E Jung DY Kim JH Patel PR Hu X Lee Y . Transient receptor potential vanilloid type-1 channel regulates diet-induced obesity, insulin resistance, and leptin resistance. FASEB J. (2015) 29:3182–92. doi: 10.1096/fj.14-268300, 25888600 PMC4511197

[53] Ye J. Mechanisms of insulin resistance in obesity. Front Med. (2013) 7:14–24. doi: 10.1007/s11684-013-0262-6, 23471659 PMC3936017

[54] Krishnan V Baskaran P Thyagarajan B. Troglitazone activates TRPV1 and causes deacetylation of PPARγ in 3T3-L1 cells. Biochim Biophys Acta Mol basis Dis. (2019) 1865:445–53. doi: 10.1016/j.bbadis.2018.11.004, 30496795 PMC6364553

[55] Gkrinia EMM Belančić A. The mechanisms of chronic inflammation in obesity and potential therapeutic strategies: a narrative review. Curr Issues Mol Biol. (2025) 47. doi: 10.3390/cimb47050357, 40699756 PMC12110701

[56] Baker RG Hayden MS Ghosh S. NF-κB, inflammation, and metabolic disease. Cell Metab. (2011) 13:11–22. doi: 10.1016/j.cmet.2010.12.008, 21195345 PMC3040418

[57] Hayden MS Ghosh S. Shared principles in NF-kappaB signaling. Cell. (2008) 132:344–62. doi: 10.1016/j.cell.2008.01.020, 18267068

[58] Zhou C Tabb MM Nelson EL Grün F Verma S Sadatrafiei A . Mutual repression between steroid and xenobiotic receptor and NF-kappaB signaling pathways links xenobiotic metabolism and inflammation. J Clin Invest. (2006) 116:2280–9. doi: 10.1172/jci26283 16841097 PMC1501109

[59] Israël A. The IKK complex, a central regulator of NF-kappaB activation. Cold Spring Harb Perspect Biol. (2010) 2:a000158. doi: 10.1101/cshperspect.a000158, 20300203 PMC2829958

[60] Page A Navarro M Suárez-Cabrera C Bravo A Ramirez A. Context-dependent role of IKKβ in Cancer. Genes (Basel). (2017) 8. doi: 10.3390/genes8120376, 29292732 PMC5748694

[61] Nandipati KC Subramanian S Agrawal DK. Protein kinases: mechanisms and downstream targets in inflammation-mediated obesity and insulin resistance. Mol Cell Biochem. (2017) 426:27–45. doi: 10.1007/s11010-016-2878-8, 27868170 PMC5291752

[62] Gao Z Hwang D Bataille F Lefevre M York D Quon MJ . Serine phosphorylation of insulin receptor substrate 1 by inhibitor kappa B kinase complex. J Biol Chem. (2002) 277:48115–21. doi: 10.1074/jbc.M209459200 12351658

[63] Vašek D Fikarová N Marková VN Honc O Pacáková L Porubská B . Lipopolysaccharide pretreatment increases the sensitivity of the TRPV1 channel and promotes an anti-inflammatory phenotype of capsaicin-activated macrophages. J Inflamm (Lond). (2024) 21:17. doi: 10.1186/s12950-024-00391-0, 38790047 PMC11127439

[64] Avila DL Fernandes-Braga W Silva JL Santos EA Campos G Leocadio PCL . Capsaicin improves systemic inflammation, atherosclerosis, and macrophage-derived foam cells by stimulating PPAR gamma and TRPV1 receptors. Nutrients. (2024) 16. doi: 10.3390/nu16183167, 39339767 PMC11435000

[65] Kang JH Tsuyoshi G Le Ngoc H Kim HM Tu TH Noh HJ . Dietary capsaicin attenuates metabolic dysregulation in genetically obese diabetic mice. J Med Food. (2011) 14:310–5. doi: 10.1089/jmf.2010.1367, 21332406

[66] Wikan N Tocharus J Oka C Sivasinprasasn S Chaichompoo W Suksamrarn A . The capsaicinoid nonivamide suppresses the inflammatory response and attenuates the progression of steatosis in a NAFLD-rat model. J Biochem Mol Toxicol. (2023) 37:e23279. doi: 10.1002/jbt.23279, 36541345

[67] Zhang CH Xiang HX Wang XW Xiao H Wei FJ Yao JC . Mechanism of Yuzhi Zhixue granules in treating polycystic ovary syndrome with insulin resistance in rats via metabolomics and proteomics. Zhongguo Zhong Yao Za Zhi. (2025) 50:3368–76. doi: 10.19540/j.cnki.cjcmm.20250304.401, 40686114

[68] Azhar Y Parmar A Miller CN Samuels JS Rayalam S. Phytochemicals as novel agents for the induction of browning in white adipose tissue. Nutr Metab (Lond). (2016) 13:89. doi: 10.1186/s12986-016-0150-6, 27980598 PMC5135798

[69] Arce-Rodríguez ML Ochoa-Alejo N. Biochemistry and molecular biology of capsaicinoid biosynthesis: recent advances and perspectives. Plant Cell Rep. (2019) 38:1017–30. doi: 10.1007/s00299-019-02406-0, 30941502

[70] Bu XW Hao XH Zhang RY Zhang MZ Wang Z Wang HS . Mechanism of Qingrun decoction in alleviating hepatic insulin resistance in type 2 diabetic rats based on amino acid metabolism reprogramming pathways. Zhongguo Zhong Yao Za Zhi. (2025) 50:3377–88. doi: 10.19540/j.cnki.cjcmm.20250120.701, 40686115

[71] Payab M Hasani-Ranjbar S Baeeri M Rahimifard M Arjmand B Haghi-Aminjan H . Development of a novel anti-obesity compound with inhibiting properties on the lipid accumulation in 3T3-L1 adipocytes. Iran Biomed J. (2020) 24:155–63. doi: 10.29252/ibj.24.3.155, 31952433 PMC7275626

[72] Mosqueda-Solís A Sánchez J Reynés B Palou M Portillo MP Palou A . Hesperidin and capsaicin, but not the combination, prevent hepatic steatosis and other metabolic syndrome-related alterations in western diet-fed rats. Sci Rep. (2018) 8:15100. doi: 10.1038/s41598-018-32875-4, 30305645 PMC6180094

[73] Chung HS Choi KM. Organokines in disease. Adv Clin Chem. (2020) 94:261–321. doi: 10.1016/bs.acc.2019.07.012, 31952573

[74] Wittwer J Bradley D. Clusterin and its role in insulin resistance and the cardiometabolic syndrome. Front Immunol. (2021) 12:612496. doi: 10.3389/fimmu.2021.612496, 33717095 PMC7946829

[75] Ahmad MI Shapiro MD. Preventing diabetes and atherosclerosis in the cardiometabolic syndrome. Curr Atheroscler Rep. (2021) 23:16. doi: 10.1007/s11883-021-00913-8, 33686460

[76] Chen H Li N Zhan X Zheng T Huang X Chen Q . Capsaicin protects against lipopolysaccharide-induced acute lung injury through the HMGB1/NF-κB and PI3K/AKT/mTOR pathways. J Inflamm Res. (2021) 14:5291–304. doi: 10.2147/JIR.S309457, 34703269 PMC8524366

[77] Zhao X Dong B Friesen M Liu S Zhu C Yang C. Capsaicin attenuates lipopolysaccharide-induced inflammation and barrier dysfunction in intestinal porcine epithelial cell line-J2. Front Physiol. (2021) 12:715469. doi: 10.3389/fphys.2021.715469, 34630139 PMC8497985

[78] Zheng Y Chen J Wu X Zhang X Hu C Kang Y . Enhanced anti-inflammatory effects of silibinin and capsaicin combination in lipopolysaccharide-induced RAW264.7 cells by inhibiting NF-κB and MAPK activation. Front Chem. (2022) 10:934541. doi: 10.3389/fchem.2022.934541, 35844639 PMC9279934

[79] Li J Wang H Zhang L An N Ni W Gao Q . Capsaicin affects macrophage anti-inflammatory activity via the MAPK and NF-κB signaling pathways. Int J Vitam Nutr Res. (2021) 93:289–97. doi: 10.1024/0300-9831/a000721 34235954

[80] Yang J Li W Wang Y. Capsaicin reduces obesity by reducing chronic low-grade inflammation. Int J Mol Sci. (2024) 25. doi: 10.3390/ijms25168979, 39201665 PMC11354495

[81] Thongin S Den-Udom T Uppakara K Sriwantana T Sibmooh N Laolob T . Beneficial effects of capsaicin and dihydrocapsaicin on endothelial inflammation, nitric oxide production and antioxidant activity. Biomed Pharmacother. (2022) 154:113521. doi: 10.1016/j.biopha.2022.113521, 36007275

[82] Saha K Sarkar D Khan U Karmakar BC Paul S Mukhopadhyay AK . Capsaicin inhibits inflammation and gastric damage during H pylori infection by targeting NF-kB-miRNA Axis. Pathogens. (2022) 11. doi: 10.3390/pathogens11060641, 35745495 PMC9227394

[83] Ahmed RA Alam MF Alshahrani S Jali AM Qahl AM Khalid M . Capsaicin ameliorates the cyclophosphamide-induced cardiotoxicity by inhibiting free radicals generation, inflammatory cytokines, and apoptotic pathway in rats. Life (Basel). (2023) 13. doi: 10.3390/life13030786, 36983940 PMC10056591

[84] Huang W Rubinstein J Prieto AR Thang LV Wang DH. Transient receptor potential vanilloid gene deletion exacerbates inflammation and atypical cardiac remodeling after myocardial infarction. Hypertension. (2009) 53:243–50. doi: 10.1161/HYPERTENSIONAHA.108.118349, 19114647 PMC2669745

[85] Li Y Guo X Zhan P Huang S Chen J Zhou Y . TRPV1 regulates proinflammatory properties of M1 macrophages in periodontitis via NRF2. Inflammation. (2024) 47:2041–56. doi: 10.1007/s10753-024-02024-3, 38700791

[86] Lv Z Xu X Sun Z Yang YX Guo H Li J . TRPV1 alleviates osteoarthritis by inhibiting M1 macrophage polarization via ca(2+)/CaMKII/Nrf2 signaling pathway. Cell Death Dis. (2021) 12:504. doi: 10.1038/s41419-021-03792-8, 34006826 PMC8131608

[87] Zhang Z Leng Z Kang L Yan X Shi J Ji Y . Alcohol inducing macrophage M2b polarization in colitis by modulating the TRPV1-MAPK/NF-kappaB pathways. Phytomedicine. (2024) 130:155580. doi: 10.1016/j.phymed.2024.155580 38810558

[88] Zhang K Qiao T Yin L Huang J Geng Z Zuo L . Pinostrobin targets the PI3K/AKT/CCL(2) axis in intestinal epithelial cells to inhibit intestinal macrophage infiltration and alleviate dextran sulfate sodium-induced colitis in mice. Nan Fang Yi Ke Da Xue Xue Bao. (2025) 45:2199–209. doi: 10.12122/j.issn.1673-4254.2025.10.16, 41139450 PMC12568474

[89] Long W Fatehi M Soni S Panigrahi R Philippaert K Yu Y . Vitamin D is an endogenous partial agonist of the transient receptor potential vanilloid 1 channel. J Physiol. (2020) 598:4321–38. doi: 10.1113/JP279961 32721035 PMC7589233

[90] Southall MD Li T Gharibova LS Pei Y Nicol GD Travers JB. Activation of epidermal vanilloid receptor-1 induces release of proinflammatory mediators in human keratinocytes. J Pharmacol Exp Ther. (2003) 304:217–22. doi: 10.1124/jpet.102.040675, 12490594

[91] Kaewpitak A Bauer CS Seward EP Boissonade FM Douglas CWI. *Porphyromonas gingivalis* lipopolysaccharide rapidly activates trigeminal sensory neurons and may contribute to pulpal pain. Int Endod J. (2020) 53:846–58. doi: 10.1111/iej.13282, 32058593

[92] Devesa I Ferrandiz-Huertas C Mathivanan S Wolf C Lujan R Changeux JP . αCGRP is essential for algesic exocytotic mobilization of TRPV1 channels in peptidergic nociceptors. Proc Natl Acad Sci USA. (2014) 111:18345–50. doi: 10.1073/pnas.1420252111, 25489075 PMC4280602

[93] Baliu-Pique M Jusek G Holzmann B. Neuroimmunological communication via CGRP promotes the development of a regulatory phenotype in TLR4-stimulated macrophages. Eur J Immunol. (2014) 44:3708–16. doi: 10.1002/eji.201444553 25316186

[94] Bulut K Felderbauer P Deters S Hoeck K Schmidt-Choudhury A Schmidt WE . Sensory neuropeptides and epithelial cell restitution: the relevance of SP- and CGRP-stimulated mast cells. Int J Color Dis. (2008) 23:535–41. doi: 10.1007/s00384-008-0447-7, 18274763

[95] Lambrecht BN. Immunologists getting nervous: neuropeptides, dendritic cells and T cell activation. Respir Res. (2001) 2:133–8. doi: 10.1186/rr49, 11686876 PMC2002076

[96] Abdel-Salam OME Mozsik G. Capsaicin, the Vanilloid receptor TRPV1 agonist in neuroprotection: mechanisms involved and significance. Neurochem Res. (2023) 48:3296–315. doi: 10.1007/s11064-023-03983-z, 37493882 PMC10514110

[97] Zhang Q Luo P Xia F Tang H Chen J Zhang J . Capsaicin ameliorates inflammation in a TRPV1-independent mechanism by inhibiting PKM2-LDHA-mediated Warburg effect in sepsis. Cell Chem Biol. (2022) 29:1248–1259 e6. doi: 10.1016/j.chembiol.2022.06.011, 35858615

[98] Yu Q Liu C Zhong X Ruo Z Jiang Q. Value of activation level of transient receptor potential cation channel subfamily V member 1 for inflammatory degree and prognosis in coronary heart disease patients combined with diabetes. Chin Heart J. (2025) 37:543–8. doi: 10.12125/j.chj.202406092

[99] Chaudhary A Gour JK Rizvi SI. Capsaicin has potent anti-oxidative effects in vivo through a mechanism which is non-receptor mediated. Arch Physiol Biochem. (2022) 128:141–7. doi: 10.1080/13813455.2019.1669056, 31566018

[100] Azlan A Sultana S Huei CS Razman MR. Antioxidant, anti-obesity, nutritional and other beneficial effects of different chili pepper: a review. Molecules. (2022) 27. doi: 10.3390/molecules27030898, 35164163 PMC8839052

[101] Liu P Guo W Gao X Yuwen M Liu Z Tan R . Capsaicin acts as a novel NRF2 agonist to suppress ethanol induced gastric mucosa oxidative damage by directly disrupting the KEAP1-NRF2 interaction. eLife. (2024). doi: 10.7554/eLife.97632.1 PMC13249423 42262974

[102] Li H Wu Z Yu B Chen J Yang C Guo Y . Dietary capsaicin supplementation mitigates calving-induced stress and enhances antioxidant capacity, immune function, and gut microbiota in periparturient dairy cows. Antioxidants (Basel). (2024) 14. doi: 10.3390/antiox14010028, 39857362 PMC11762672

[103] Yang Z Guo H Zhang P Liu K Ba J Bai X . Capsaicin (CAP) exerts a protective effect against ethanol-induced oxidative gastric mucosal injury by modulating the chemokine receptor 4 (CCR4)/Src/p47phox signaling pathway both in vitro and in vivo. Chin J Nat Med. (2025) 23:191–202. doi: 10.1016/S1875-5364(25)60823-5, 39986695

[104] Dludla PV Cirilli I Marcheggiani F Silvestri S Orlando P Muvhulawa N . Bioactive properties, bioavailability profiles, and clinical evidence of the potential benefits of black pepper (*Piper nigrum*) and red pepper (Capsicum annum) against diverse metabolic complications. Molecules. (2023) 28. doi: 10.3390/molecules28186569, 37764345 PMC10534530

[105] Karimi-Sales E Mohaddes G Alipour MR. Hepatoprotection of capsaicin in alcoholic and non-alcoholic fatty liver diseases. Arch Physiol Biochem. (2024) 130:38–48. doi: 10.1080/13813455.2021.1962913, 34396890

[106] Wang Y Cui L Xu H Liu S Zhu F Yan F . TRPV1 agonism inhibits endothelial cell inflammation via activation of eNOS/NO pathway. Atherosclerosis. (2017) 260:13–9. doi: 10.1016/j.atherosclerosis.2017.03.016, 28324760

[107] Yang C Guo W He R Meng X Fu J Lu Y. Dietary capsaicin attenuates cardiac injury after myocardial infarction in type 2 diabetic mice by inhibiting ferroptosis through activation of TRPV1 and Nrf2/HMOX1 pathway. Int Immunopharmacol. (2024) 140:112852. doi: 10.1016/j.intimp.2024.112852 39106715

[108] Sun J Pu Y Wang P Chen S Zhao Y Liu C . TRPV1-mediated UCP2 upregulation ameliorates hyperglycemia-induced endothelial dysfunction. Cardiovasc Diabetol. (2013) 12:69. doi: 10.1186/1475-2840-12-69 23607427 PMC3644255

[109] Segawa Y Hashimoto H Maruyama S Shintani M Ohno H Nakai Y . Dietary capsaicin-mediated attenuation of hypertension in a rat model of renovascular hypertension. Clin Exp Hypertens. (2020) 42:352–9. doi: 10.1080/10641963.2019.1665676, 31518162

[110] Yang J Du M Zhang L Xu X. High dose capsaicinoid increases the susceptibility to DSS-induced colitis through ROS/NFκB/NLRP3 pathway and mitophagy disorder by calcium overload in macrophages. Food Biosci. (2025) 65. doi: 10.1016/j.fbio.2025.106084

[111] Gordon L Buncke HJ. Heterotopic free skeletal muscle autotransplantation with utilization of a long nerve graft and microsurgical techniques: a study in the primate. J Hand Surg Am. (1979) 4:103–8. doi: 10.1016/s0363-5023(79)80125-2, 106084

[112] Wu F Bu S Wang H. Role of TRP channels in metabolism-related diseases. Int J Mol Sci. (2024) 25. doi: 10.3390/ijms25020692, 38255767 PMC10815096

[113] Kawada T Hagihara K Iwai K. Effects of capsaicin on lipid metabolism in rats fed a high fat diet. J Nutr. (1986) 116:1272–8. doi: 10.1093/jn/116.7.1272, 2875141

[114] Shin MK Yang SM Han IS. Capsaicin suppresses liver fat accumulation in high-fat diet-induced NAFLD mice. Anim Cells Syst (Seoul). (2020) 24:214–9. doi: 10.1080/19768354.2020.1810771, 33029298 PMC7473188

[115] Mun JM Ok HM Kwon O. Corn gluten hydrolysate and capsaicin have complimentary actions on body weight reduction and lipid-related genes in diet-induced obese rats. Nutr Res. (2014) 34:458–65. doi: 10.1016/j.nutres.2014.04.009, 24916560

[116] Rohm B Riedel A Ley JP Widder S Krammer GE Somoza V. Capsaicin, nonivamide and trans-pellitorine decrease free fatty acid uptake without TRPV1 activation and increase acetyl-coenzyme a synthetase activity in Caco-2 cells. Food Funct. (2015) 6:173–85. doi: 10.1039/c4fo00435c, 25422952

[117] Uddandrao VVS Chandrasekaran P Saravanan G Brahmanaidu P Sengottuvelu S Ponmurugan P . Phytoformulation with hydroxycitric acid and capsaicin protects against high-fat-diet-induced obesity cardiomyopathy by reducing cardiac lipid deposition and ameliorating inflammation and apoptosis in the heart. J Tradit Complement Med. (2024) 14:162–72. doi: 10.1016/j.jtcme.2023.08.004, 38481548 PMC10927456

[118] Panchal SK Bliss E Brown L. Capsaicin in metabolic syndrome. Nutrients. (2018) 10:5. doi: 10.3390/nu10050630, 29772784 PMC5986509

[119] Zhao Y Li X Tian Y Zhao J Yu W Zhang L . Nonivamide induces brown fat-like characteristics in porcine subcutaneous adipocytes. Biochem Biophys Res Commun. (2022) 619:68–75. doi: 10.1016/j.bbrc.2022.06.047, 35738067

[120] Gong T Wang H Liu S Zhang M Xie Y Liu X. Capsaicin regulates lipid metabolism through modulation of bile acid/gut microbiota metabolism in high-fat-fed SD rats. Food Nutr Res. (2022) 66. doi: 10.29219/fnr.v66.8289 PMC9180124 35721805

[121] Parks DJ Blanchard SG Bledsoe RK Chandra G Consler TG Kliewer SA . Bile acids: natural ligands for an orphan nuclear receptor. Science. (1999) 284:1365–8. doi: 10.1126/science.284.5418.1365, 10334993

[122] Sha X Lin J Wu K Lu J Yu Z. The TRPV1-PKM2-SREBP1 axis maintains microglial lipid homeostasis in Alzheimer's disease. Cell Death Dis. (2025) 16:14. doi: 10.1038/s41419-024-07328-8, 39809738 PMC11732990

[123] Ma L Zhong J Zhao Z Luo Z Ma S Sun J . Activation of TRPV1 reduces vascular lipid accumulation and attenuates atherosclerosis. Cardiovasc Res. (2011) 92:504–13. doi: 10.1093/cvr/cvr245, 21908651

[124] Zhao JF Ching LC Kou YR Lin SJ Wei J Shyue SK . Activation of TRPV1 prevents OxLDL-induced lipid accumulation and TNF-α-induced inflammation in macrophages: role of liver X receptor α. Mediat Inflamm. (2013) 2013:925171. doi: 10.1155/2013/925171, 23878415 PMC3710635

[125] Gong T Li C Li S Zhang X He Z Jiang X . Capsaicin regulates dyslipidemia by altering the composition of bile acids in germ-free mice. Food Funct. (2022) 13:10665–79. doi: 10.1039/d2fo02209e, 36172720

[126] Yang Q La C Prabahar K Safargar M Kord-Varkaneh H Temuqile . The effect of capsaicin, capsinoids, and pepper-based interventions on lipid profiles in overweight or obese individuals: a systematic review and meta-analysis of randomized controlled trials. Diabetes Res Clin Pract. (2025) 229:112478. doi: 10.1016/j.diabres.2025.112478 40939866

[127] Gong T Zhou Y Zhang L Wang H Zhang M Liu X. Capsaicin combined with dietary fiber prevents high-fat diet associated aberrant lipid metabolism by improving the structure of intestinal flora. Food Sci Nutr. (2023) 11:114–25. doi: 10.1002/fsn3.3043, 36655087 PMC9834886

[128] Wang ZH Du WW Qian FY Hou HY Le Deng J Ren XR . Homocapsaicin II induce ferroptosis in colorectal cancer cells via cholesterol-centrosome amplification-multipolarity axis. J Ethnopharmacol. (2025) 348:119894. doi: 10.1016/j.jep.2025.119894, 40319933

[129] Yang D Luo Z Ma S Wong WT Ma L Zhong J . Activation of TRPV1 by dietary capsaicin improves endothelium-dependent vasorelaxation and prevents hypertension. Cell Metab. (2010) 12:130–41. doi: 10.1016/j.cmet.2010.05.015, 20674858 PMC3906919

[130] Chen Q Zhu H Zhang Y Zhang Y Wang L Zheng L. Vasodilating effect of capsaicin on rat mesenteric artery and its mechanism. Zhejiang Da Xue Xue Bao Yi Xue Ban. (2013) 42:177–83. doi: 10.3785/j.issn.1008-9292.2013.02.008, 23585004

[131] Amssayef A Eddouks M. Alkaloids as vasodilator agents: a review. Curr Pharm Des. (2023) 29:1886–95. doi: 10.2174/1381612829666230809094313, 37559238

[132] Varela-López E Del Valle-Mondragón L Castrejón-Téllez V Pérez-Torres I Arenas AP Rojas FM . Role of the transient receptor potential vanilloid type 1 (TRPV1) in the regulation of nitric oxide release in Wistar rat aorta. Oxidative Med Cell Longev. (2021) 2021:8531975. doi: 10.1155/2021/8531975, 34394835 PMC8355966

[133] Zhang L Lu W Lu C Guo Y Chen X Chen J . Beneficial effect of capsaicin via TRPV4/EDH signals on mesenteric arterioles of normal and colitis mice. J Adv Res. (2022) 39:291–303. doi: 10.1016/j.jare.2021.11.001, 35777913 PMC9263647

[134] Juan L Qian-Hui S. A6705 inhibitory effects of capsaicin on the proliferation of rat vascular smooth muscle cells induced by high sodium. J Hypertens. (2018) 36:e19. doi: 10.1097/01.hjh.0000548062.27801.c2

[135] Zhao S Liu W Feng C Zhang X Cai W Luo M. Effect and molecular mechanisms of collateral vessel growth mediated by activation of transient receptor potential Vanilloid type 1. J Vasc Res. (2020) 57:185–94. doi: 10.1159/000506516, 32526735

[136] Leroux A Roque M Casas E Leng J Guibert C L'Azou B . The effect of CGRP and SP and the cell signaling dialogue between sensory neurons and endothelial cells. Biol Res. (2024) 57:65. doi: 10.1186/s40659-024-00538-6 39261966 PMC11389267

[137] Zaleski K Matias A Gyampo A Giuriato G Lynch M Lora B . Does sex influence near-infrared spectroscopy-derived indicators of microvascular reactivity and the response to acute dietary capsaicin. Microvasc Res. (2023) 145:104436. doi: 10.1016/j.mvr.2022.104436, 36113667

[138] Kumar V Kumar V Mahajan N Kaur J Devi K Dharavath RN . Mucin secretory action of capsaicin prevents high fat diet-induced gut barrier dysfunction in C57BL/6 mice colon. Biomed Pharmacother. (2022) 145:112452. doi: 10.1016/j.biopha.2021.112452, 34808551

[139] Zhang X Hu H Zhang Y Hu S Lu J Peng W . Dietary capsaicin exacerbates gut microbiota Dysbiosis and mental disorders in type 1 diabetes mice. Nutrients. (2025) 17. doi: 10.3390/nu17030593, 39940450 PMC11821225

[140] Dai Z Li S Meng Y Zhao Q Zhang Y Suonan Z . Capsaicin ameliorates high-fat diet-induced atherosclerosis in ApoE(−/−) mice via remodeling gut microbiota. Nutrients. (2022) 14. doi: 10.3390/nu14204334, 36297020 PMC9611743

[141] Rosca AE Iesanu MI Zahiu CDM Voiculescu SE Paslaru AC Zagrean AM. Capsaicin and gut microbiota in health and disease. Molecules. (2020) 25. doi: 10.3390/molecules25235681, 33276488 PMC7730216

[142] Wang Y Tang C Tang Y Yin H Liu X. Capsaicin has an anti-obesity effect through alterations in gut microbiota populations and short-chain fatty acid concentrations. Food Nutr Res. (2020) 64. doi: 10.29219/fnr.v64.3525 PMC7054644 32180694

[143] Shen W Shen M Zhao X Zhu H Yang Y Lu S . Anti-obesity effect of capsaicin in mice fed with high-fat diet is associated with an increase in population of the gut bacterium *Akkermansia muciniphila*. Front Microbiol. (2017) 8:272. doi: 10.3389/fmicb.2017.00272, 28280490 PMC5322252

[144] Gong T Zhou Y Shi Q Li Y Wang H Zhang M . Capsaicin modulates *Akkermansia muciniphila* abundance by enhancing MUCIN2 levels in mice fed with high-fat diets. Food Nutr Res. (2023) 67. doi: 10.29219/fnr.v67.9990 PMC11077401 38721112

[145] Jia ET Liu ZY Pan M Lu JF Ge QY. Regulation of bile acid metabolism-related signaling pathways by gut microbiota in diseases. J Zhejiang Univ Sci B. (2019) 20:781–92. doi: 10.1631/jzus.B1900073, 31489798 PMC6751489

[146] Mahalak KK Bobokalonov J Firrman J Williams R Evans B Fanelli B . Analysis of the ability of capsaicin to modulate the human gut microbiota in vitro. Nutrients. (2022) 14. doi: 10.3390/nu14061283, 35334939 PMC8950947

[147] Li J Liao X Yin X Deng Z Hu G Zhang W . Gut microbiome and serum metabolome profiles of capsaicin with cognitive benefits in APP/PS1 mice. Nutrients. (2022) 15. doi: 10.3390/nu15010118, 36615777 PMC9823564

[148] Xia Y Lee G Yamamoto M Takahashi H Kuda T. Detection of indigenous gut bacteria related to red chilli pepper (*Capsicum annuum*) in murine caecum and human faecal cultures. Mol Biol Rep. (2022) 49:10239–50. doi: 10.1007/s11033-022-07875-3, 36068389

[149] Cheng P Wu J Zong G Wang F Deng R Tao R . Capsaicin shapes gut microbiota and pre-metastatic niche to facilitate cancer metastasis to liver. Pharmacol Res. (2023) 188:106643. doi: 10.1016/j.phrs.2022.106643, 36608780

[150] Deng R Yu S Ruan X Liu H Zong G Cheng P . Capsaicin orchestrates metastasis in gastric cancer via modulating expression of TRPV1 channels and driving gut microbiota disorder. Cell Commun Signal. (2023) 21:364. doi: 10.1186/s12964-023-01265-3, 38129926 PMC10734064

[151] Xiang Q Tang X Cui S Zhang Q Liu X Zhao J . Capsaicin, the spicy ingredient of chili peppers: effects on gastrointestinal tract and composition of gut microbiota at various dosages. Foods. (2022) 11. doi: 10.3390/foods11050686, 35267319 PMC8909049

[152] Kang C Wang B Kaliannan K Wang X Lang H Hui S . Gut microbiota mediates the protective effects of dietary capsaicin against chronic low-grade inflammation and associated obesity induced by high-fat diet. MBio. (2017) 8. doi: 10.1128/mBio.00470-17, 28536285 PMC5442453

[153] Li W Sun X Wang J Zhao Q Dai R Wang Y . Defective thymic output in WAS patients is associated with abnormal actin organization. Sci Rep. (2017) 7:11978. doi: 10.1038/s41598-017-12345-z, 28931895 PMC5607224

[154] Kimura I Ozawa K Inoue D Imamura T Kimura K Maeda T . The gut microbiota suppresses insulin-mediated fat accumulation via the short-chain fatty acid receptor GPR43. Nat Commun. (2013) 4:1829. doi: 10.1038/ncomms2852, 23652017 PMC3674247

[155] Ren M Xia Y Pan H Zhou X Yu M Ji F. Duodenal-jejunal bypass ameliorates MASLD in rats by regulating gut microbiota and bile acid metabolism through FXR pathways. Hepatol Commun. (2025) 9. doi: 10.1097/hc9.0000000000000615 PMC11737483 39813598

[156] Wan J Lang C Gao M Liu F Feng X Li H . Schisandrin B alleviates metabolic associated fatty liver disease by regulating the PPARγ signaling pathway and gut microbiota in mice. Front Pharmacol. (2025) 16:1583307. doi: 10.3389/fphar.2025.1583307, 40786039 PMC12331688

[157] Fan X Xiao Z Chen Y Yang H Diao M Hu W . Interactions between gut microbiota and Parkinson’s disease: the role of tryptophan metabolism. Cell Commun Signal. (2025) 23:424. doi: 10.1186/s12964-025-02393-8, 41068876 PMC12512717

[158] Kim HJ. Capsaicin supplementation prevents western diet-induced hyperleptinemia by reducing endoplasmic reticulum stress in apolipoprotein E-deficient mice. Food Nutr Res. (2023) 67. doi: 10.29219/fnr.v67.9610 PMC10710870 38084147

[159] Wang Y Zhou Y Fu J. Advances in antiobesity mechanisms of capsaicin. Curr Opin Pharmacol. (2021) 61:1–5. doi: 10.1016/j.coph.2021.08.012, 34537583

[160] Kang ZQ Hu JL Chen MY Mao Y Xie LF Yang N . Effects of capsaicin on the hypoglycemic regulation of metformin and gut microbiota profiles in type 2 diabetic rats. Am J Chin Med. (2022) 50:839–61. doi: 10.1142/s0192415x22500355, 35300567

